# Biophysical modeling of thalamic reticular nucleus subpopulations and their differential contribution to spindle dynamics

**DOI:** 10.1016/j.isci.2025.113393

**Published:** 2025-08-20

**Authors:** Polina Litvak, Nolan D. Hartley, Ryan Kast, Guoping Feng, Zhanyan Fu, Alexis Arnaudon, Sean L. Hill

**Affiliations:** 1 Blue Brain Project, École Polytechnique Fédérale de Lausanne (EPFL), Campus Biotech, Geneva, Switzerland; 2 Stanley Center for Psychiatric Research, Broad Institute of MIT and Harvard, Cambridge, MA 02142, USA

**Keywords:** Systems neuroscience, Cellular neuroscience, Computational bioinformatics

## Abstract

Burst firing in thalamic reticular neurons is key to sleep rhythms and is linked to neurodevelopmental disorders. Several models of reticular neurons are currently available; however, a biophysically detailed model reproducing experimental burst firing heterogeneity is lacking. We addressed this by combining patch-clamp electrophysiology of fluorescently tagged Spp1+ and Ecel1+ neurons with a previously established statistical framework to differentiate cell types. We developed a population of biophysically detailed thalamic reticular models capturing diverse firing properties, particularly varied rebound bursting. These models incorporate key ion channels, such as T-type Ca^2+^ and small conductance potassium channels (SK), allowing systematic study of their impact on single-cell dynamics. By integrating these models into a thalamic microcircuit, we demonstrate that T-type Ca^2+^ and SK channel conductances have opposing effects on spindle oscillations. We identify a simple relationship between these conductances and spindle peak firing frequency, and provide a foundation for relating cellular properties to network activity.

## Highlights

• Developed computational models of diverse thalamic reticular nucleus (TRN) neurons
• Integrated models into a thalamic microcircuit to study TRN network dynamics
• Model predicts a link between TRN makeup, dendritic T-type Ca^2+^, and spindle oscillations
• Platform enables study of TRN function and dysfunction in health and disease

## Graphical abstract

**Figure:**
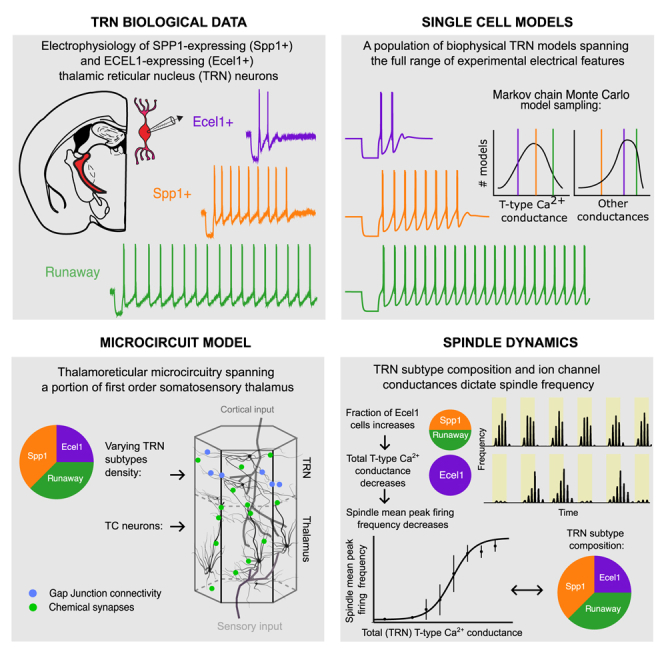

## Introduction

The thalamic reticular nucleus (TRN) is a shell-like subcortical structure surrounding the dorsal thalamus. It is composed of a heterogeneous population of gamma-aminobutyric acid (GABA)-ergic neurons and provides a major source of inhibition to the thalamus in rodents.^1^ Upon stimulation, reticular neurons generate action potentials (APs) in two distinct firing modes, tonic, and burst, depending on their membrane potential and their expression of low-threshold voltage-gated T-type ${\text{Ca}}^{2+}$ channels.^2^ At resting or relatively depolarized membrane potentials, reticular neurons fire regular tonic sodium spikes. At hyperpolarized potentials, they generate repetitive low-threshold calcium transients superimposed with high-frequency sodium spikes, referred to as bursts. TRN bursts are efficient at producing postsynaptic inhibition, while rebound bursting in both TRN and thalamic relay cells plays a crucial role in generating and maintaining thalamocortical rhythms.^3^ Multiple previous studies establish the reticular thalamus as the origin of spindle activity, a major brain oscillation most prevalent in non-rapid eye movement (NREM) sleep, further regulated by thalamocortical interactions.^3^^,^^4^^,^^5^ In naturally sleeping rodents, the duration and frequency of spindle oscillations are shown to be directly shaped by reticular inhibition.^6^ Building upon these experimental findings, a recent computational thalamoreticular circuit model was able to link spindle duration to membrane potential dynamics in both thalamic relay and reticular neurons.^7^

The distinct firing properties of TRN neurons critically depend on the subtypes of ion channels they express. A large body of literature identifies the T-type low threshold calcium channel family as a key mediator of burst firing in these neurons.^2^^,^^8^ However, the specific roles of different T-type channel isoforms in TRN rhythm generation, and how they contribute to the variable bursting propensity of reticular cells, are not fully elucidated. Furthermore, TRN bursts are typically followed by an afterhyperpolarization (AHP) generated by small-conductance calcium-activated (SK)-type potassium currents. The precise interplay between these SK channels and T-type ${\text{Ca}}^{2+}$ channels, particularly in shaping rebound burst activity within specific TRN cell subpopulations, represents a significant knowledge gap, which this work aims to address.

TRN cells greatly vary in their propensity to generate bursts.^2^^,^^9^^,^^10^ Two recent studies, using immunohistochemical staining for molecular markers^11^ as well as transcriptomics^8^ give an unprecedented characterization of reticular cells in terms of cellular morphology, molecular expression, axonal connectivity, and physiological activity. According to their multiscale single-cell analysis, the heterogeneity of TRN cells is characterized by a transcriptomic gradient composed of two negatively correlated gene expression profiles. Neurons at the extremes of the gradient have a near-exclusive expression of a few marker genes used to segregate them into a core (Spp1+) and shell (Ecel1+) TRN subpopulation with a differential propensity to generate intrinsic rebound bursting^8^ and consequently a differential contribution to network dynamics, as well as distinct roles in neuropsychiatric disorders like epilepsy and schizophrenia. TRN neurons that exhibit strong rebound bursting dynamics mediated by large T-type Ca currents such as parvalbumin-expressing and Spp1+ neurons, are crucial for generating thalamocortical rhythms and processing sensory information. Disruptions in these TRN subpopulations may lead to disorders with excessive rhythmogenesis, such as epileptic seizures.^9^ Conversely, dysfunction in somatostatin-positive and Ecel1+ neurons, endowed with smaller T-currents and lower frequency bursts, may contribute to the cognitive and emotional symptoms associated with schizophrenia.^9^^,^^12^

Although several computational models of reticular neurons have been developed to date,^13^^,^^14^ a biophysically accurate model capable of effectively capturing the diverse continuum of TRN electrical properties and replicating the heterogeneity of TRN rebound burst firing remained unavailable. Using data obtained from novel Cre mouse lines targeting genetically segregated TRN populations expressing the Spp1 and Ecel1 genes,^12^ and leveraging the Markov chain Monte Carlo (MCMC) approach of Arnaudon et al.,^15^ we built electrical reticular models of the thalamus that reproduce the full range of firing modes observed experimentally.

We then incorporated these models into the thalamoreticular microcircuit of Iavarone et al.,^7^ which consists of detailed three-dimensional morphological reconstructions that constrain the precise connectivity between neurons of the thalamus and the reticular nucleus. Its synaptic connections comprise chemical synapses with short-term depression and facilitation, and electrical synapses (gap junctions) between TRN neuron dendrites, known to provide a basis for neuronal synchronization in thalamic networks. By replacing the original electrical models with our Spp1 and Ecel1 TRN models, while keeping the connectome unchanged, we could delineate the differential contributions of TRN intrinsic conductances to spindle-like activity as well as predict the relative fraction of Ecel1+ and Spp1+ in the somatosensory TRN from spindle properties under knock-down experiments of ${\text{Ca}}^{2+}$ channels similar to Li et al.^8^ These predictions and delineations provide important insights into how the intrinsic cellular properties of reticular cells shape emergent circuit dynamics, advancing our understanding of thalamic rhythmogenesis in both health and disease.

## Results

### Experimental data

**Figure 1:**
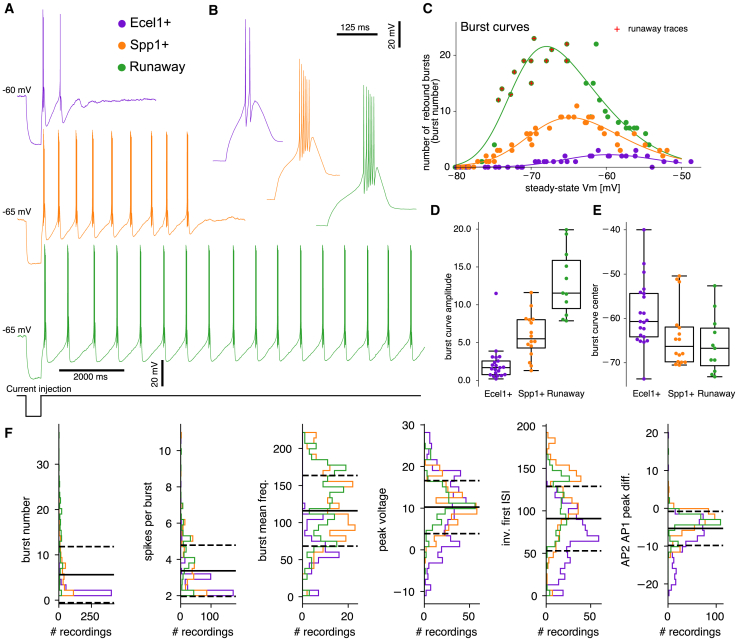
Experimental data of thalamic reticular cells (A) Exemplar traces of Ecel1+ (top), Spp1+ (middle), and Runaway (bottom) cells. The current trace represents the hyperpolarization step of 500 ms and varied amplitudes to target steady-state voltages. (B) Zoom on the first burst of each trace from (A). (C) Burst number as a function of steady-state voltage (burst curves) for Ecel1+ (left), Spp1+ (middle), runaway (right) of the cells of (A). Red cross indicates traces with runaway property, the line is a fit of a skewed Gaussian curve (see STAR Methods). (D) Distributions of the burst curve amplitude parameter (skewed Gaussian fit) by cell type. (E) Distributions of the burst curve center parameter (skewed Gaussian fit) by cell type. (F) Distributions of the main electrical features used for model building, split by cell type. The black line is the average of combined data across three cell classes and the dashed line represents the first standard deviation. Each data point is a cell trace recorded at a specific steady-state voltage (number of points are: Ecel1: 883, Spp1: 579, Runaway: 401, total: 1863).

This modeling work was based on electrophysiology data of Hartley et al.,^12^ recorded from two transcriptomically defined populations of TRN neurons, referred to as Ecel1+ and Spp1+, which represent the two genes most differentially expressed by the identified genetic profiles. The dataset consisted of whole-cell patch clamp recordings from 52 genetically labeled Spp1+ or Ecel1+ neurons, subjected to several depolarizing and hyperpolarizing current step protocols. For the protocol assessing rebound bursting activity, referred to as the burst protocol, neurons were subjected to hyperpolarizing current injections to a voltage near maximal de-inactivation of T-type ${\text{Ca}}^{2+}$ channels to induce maximal rebound bursting for each cell, starting from various holding potentials. On average, Spp1+ neurons displayed a larger number of bursts than Ecel1+ neurons, and for a subset of steady-state voltages, roughly half of the Spp1+ neurons were able to continue bursting past the 15 s sampling window (and up to several minutes) and were therefore defined as Spp1+ runaway bursting neurons.^12^ Consequently, each cell from the experimental database was classified into one of three electrical types Ecel1, Spp1, or Spp1 runaway (referred to as Runaway). Representative voltage traces of these cell subtypes are displayed in Figure 1A with a zoom on the first burst in Figure 1B, illustrating the variability in the number of APs per burst. The scan of initial steady-state voltages of the burst protocol is shown in Figure 1C for one cell per type. We referred to this as the cell’s burst curve. A skewed Gaussian function (see STAR Methods and Figure S1) was fitted to these points to extract key parameters, including curve center location (mV), amplitude, and skewness of the burst curve.

In Figures 1D and 1E we show the distribution of two parameters (see Figure S1 for the others). The burst curve amplitude was, as expected, largest for Runaway and smallest for Ecel1+ cells. Interestingly, however, Ecel1+ burst curves peaked at higher voltages than Spp1+ curves. This is consistent with the higher expression of the Cav3.1 T-type ${\text{Ca}}^{2+}$ channel subtype in Ecel1+ cells (see Figure S8), which is known to open at higher voltages^16^ than the other channel subtypes. A broader range of burst curves, highlighting the variability in their shapes, including cells with highly skewed curves or curves lacking a distinct peak, is presented in supplemental information (SI) Figure S1. This variability was observed even within the same cell type.

In addition to bursting properties, we summarized important features of the experimental data used to build our models in Figure 1F, for each cell type. These data include all recordings with bursts, in contrast to the burst curves, which used only traces with maximum elicited bursts per cell. Notably, many electrical properties were similar across cell types. However, the first inter-spike interval (ISI) was shorter for Spp1+/Runaway cells compared to Ecel1+, and this shorter ISI correlated with a higher number of APs per burst, as shown in Figures 1B and S6. As discussed later in the text, there is a significant correlation between intra-burst AP frequency, first ISI, and the number of APs per burst, which can be attributed to the level of SK current in the cell.

To further quantify how well the extracted electrical features distinguish these cell types, we employed an XGBoost feature classifier^17^ to classify cells based solely on their feature values (see Figure S3). With a 10-fold cross-validation, we obtained a classification accuracy of 89±14.7%. Omitting the burst number resulted in only a marginal drop of approximately 1% in accuracy, indicating that electrical features, such as the runaway measure (a metric tracking slow AHP depth between bursts), burst frequency, or time to first spike (the top three most important features) are highly representative of the differences in intrinsic electrophysiological properties between the three cell types.

### Building electrical models of bursting cells with Markov chain Monte Carlo

To build and study the full range of electrical models of TRN cells, and in particular, the three subtypes identified experimentally, we used the Markov chain Monte Carlo (MCMC) approach from our prior work.^15^ Instead of a direct optimization as was done previously,^18^^,^^19^ we sampled many electrical models from a probability distribution inversely proportional to a cost function, constructed by comparing electrical features of the model with experimental data. This allowed us to create many models with a low value of the cost function encompassing all subtypes we were interested in, making it possible to understand the differences between them. In particular, the distinction between Ecel1+ and Spp1+ cells was a continuum rather than a dichotomy,^8^ hence creating many models spanning the full range of electrical features obtained experimentally, rather than building one specific model per cell type, provided a more detailed understanding of these TRN cell subpopulations. In more detail, the cost function was constructed from the *Z* scores of a set of electrical features extracted from traces under specific protocols of somatic current injections (see Table S1). In principle, these values were to be extracted directly from experimental data.^15^^,^^19^^,^^20^ However, the recordings were obtained under varying steady-state voltages, and the burst curves exhibited substantial variability, making a reliable automated feature extraction method impossible. Additionally, numerically processing the numerous traces required for the complete experimental burst curves would have incurred prohibitive computational costs. Therefore, the protocol employed was comprised of the following select current steps: a holding step to maintain the model at a target voltage during the entire protocol and a 500 ms hyperpolarizing step to bring the voltage to −100 mV. This protocol included three variants with steady-state voltages of −80 mV, −65 mV, and −55 mV. The first target voltage ensured that the cell did not burst after the hyperpolarization step, while the next two ensured that it did up to at least −55 mV, as observed experimentally.

The electrical models we implemented were based on the model of Destexhe et al.,^13^ similar to the thalamic relay cell models of Iavarone et al.^18^ or more recently the TRN models of Iavarone et al.^7^ We used a detailed morphology (comprising soma and basal dendrites) derived from morphological reconstructions described in Iavarone et al.^7^ (see Figures S2 and 2A for an illustration of the morphology). We placed Na and K channels in the soma to generate action potentials (hh2_Na and hh2_K), generic T-type ${\text{Ca}}^{2+}$ channels ($I_{T}$ ), and generic SK channels ($I_{\text{AHP}}$ ) in both soma and basal dendrites to generate low-threshold ${\text{Ca}}^{2+}$ spikes. Ca^2+^ dynamics (cad) with ATPase pump and first-order buffering was also included in all compartments. We also included a ${\text{Ca}}^{2+}$-dependent nonspecific cation current ($I_{\text{CAN}}$ ) and a fast transient potassium current ($I_{A}$) in basal dendrites to control the rebound burst properties. In addition, consistent with experimental findings showing higher T-type ${\text{Ca}}^{2+}$ channel density in distal TRN neuron dendrites,^21^ we modeled a linear increase in $I_{T}$ conductance along basal dendrites, using a slope and maximum conductance as parameters (see STAR Methods and Table S2 for details). We also considered a parameter to apply a voltage shift to the T-type ${\text{Ca}}^{2+}$ ion channel model (denoted vshift_it2), to control the location of the ${\text{Ca}}^{2+}$ window current, known to be important for the bursting properties of the cells. The connection between this parameter and the relative conductances of Cav3 channel subtypes is explored later in the text.

**Figure 2:**
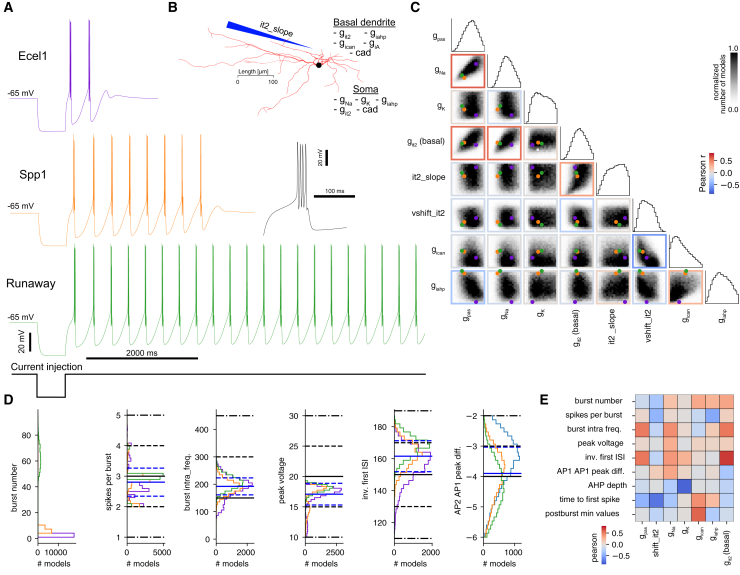
Model building of thalamic reticular cells (A) Three traces of corresponding models of each electrical type. Zoom on the first burst of Ecel1 model in the inset. (B) Detailed reconstruction of the morphology used for model building, with a list of ion channels included in each compartment (see STAR Methods and Figure S2). (C) Corner plot of densities of models with SD <2. Frames are colored with Pearson correlation between parameters, dots represent the location of the three models from (A). (D) Feature distribution of the main features used for MCMC constraints, split by cell type. The mean (black line), 1 standard deviation (SD, dashed line) and 2 SD (dot-dashed lines) are constraints used in the cost function (see SI Table S1). Blue lines are mean and 1 SD of the data, with all cell types combined. (E) Pearson correlations between select model parameters and features (same scale as in C).

We ran MCMC with bounds of model parameters adjusted to capture a large part of the parameter space that contained valid models, defined as models with maximal scores of less than 2 standard deviations (SDs). In Figure 2C, the corner plot of model densities in the parameter space shows significant correlations between many pairs of parameters (colored frames with Pearson correlations). For example, $I_{T}$ conductance in basal dendrites was positively correlated with $g_{\text{pas}}$ and somatic Na, due to the constraint imposed by experimental data on the relative amplitude of the first two action potentials (not shown). Indeed, for larger low-threshold ${\text{Ca}}^{2+}$ spikes in the basal dendrites, the soma required more Na to trigger Na spikes at a fast rate and a similar amplitude. The slope of the $I_{T}$ distribution in basal dendrites and its maximal conductance were also positively correlated, as for larger it2_slope values (smaller $I_{T}$ conductances along the dendrites, see STAR Methods), the total amount of $I_{T}$ was reduced, hence compensated with a global increase via the $I_{T}$ conductance parameter. On the contrary, $I_{\text{CAN}}$ conductance and the voltage shift parameter of the T-type channel mechanism (see STAR Methods) as well as $I_{\text{AHP}}$ and passive conductances had a negative correlation from the constraint imposed by the slow AHP depth and burst intra-frequency features (these features become large away from the sampled space of models at 2 SDs, not shown).

From the many models we obtained, we show three representatives of each cell class under the rebound burst protocol at −65mV in Figure 2A, similar to the experimental data of Figure 1A. The parameter subsets for these randomly selected models are shown as colored dots in Figure 2C and in Table S2. Notably, the primary distinction between Ecel1 and Spp1/Runaway cell types in these models was due to significant differences in $I_{\text{AHP}}$ conductance (see also Figure S7). Conversely, the parameters of Spp1 and Runaway models showed greater similarity.

In Figure 2D, we show the distributions of the main features used by MCMC, the mean and SD we used to build the cost function shown in black (see Table S1) and the mean and SD of all the samples with SD <2 highlighted in blue. We were successful in reproducing the difference in first ISI between cell types, as seen in the experimental data across a large range of steady-state voltages (see Figure 1F), or the average number of spikes per burst. The AP (peak voltage) amplitude feature in the experimental data had a large variability, we therefore chose to restrict it to high values with a mean value of 20mV, as modeling this variability was out of scope of this work. For the relative amplitude of the first two APs, we observed an opposite trend to experimental data, with Ecel1 models having a smaller decrease than the other two model types. Interestingly, the drop was larger in the experimental data, even when disregarding the long tail between −10 and −20 mV (in Figure 1F), with cells able to generate only a single, weak burst, with a strong decay of AP amplitude (not shown). This smaller drop of AP amplitudes observed in Ecel1 models was due to a lower level of $I_{T}$ conductance in basal dendrites, which was negatively correlated with this feature. This mismatch with experimental data potentially stemmed from the oversimplification of the T-type ${\text{Ca}}^{2+}$ channel kinetics modeled with a generic $I_{T}$ conductance.

In Figure 2E, we present Pearson correlations between model parameters and electrical features, thereby identifying the parameters that exerted the most significant influence on each feature. For example, $I_{T}$ conductance in basal dendrites controlled the first ISI (and the burst mean frequency, as ISI are fairly regular). Indeed, increased $I_{T}$ in basal dendrites has been demonstrated to produce larger low-threshold spikes,^22^ which, in turn, generated stronger somatic bursts of Na spikes with increased AP number and frequency. We further analyzed correlations between experimental and model features, as summarized in Figure S6. Our models successfully reproduced the most prominent correlations between the number of APs in a burst, intra-burst frequency, and the first ISI. However, due to the inherent variability of experimental burst curves and the fact that experimental features were derived from mean values across recordings with varying steady-state voltages, several correlation discrepancies were observed. Furthermore, the simplified ionic mechanisms used in our MCMC-generated models, which did not capture the nuanced differences in ion channel isoforms differentially expressed by Ecel1+ and Spp1+ neurons (see Figure S8), also contributed to these mismatches. We provide additional numerical insights into T-type isoforms later in the text.

Despite certain limitations, the MCMC approach enabled us to sample valid models for all three cell subtypes (Ecel1, Spp1, and Runaway) in a single run, avoiding the need for separate sampling runs with adjusted cost functions. This allowed us to analyze the resulting “continuum of models” and characterize their differences and similarities in terms of electrical features and intrinsic cellular mechanisms.

### Distinguishing TRN electrical subtypes from model features and parameters

**Figure 3:**
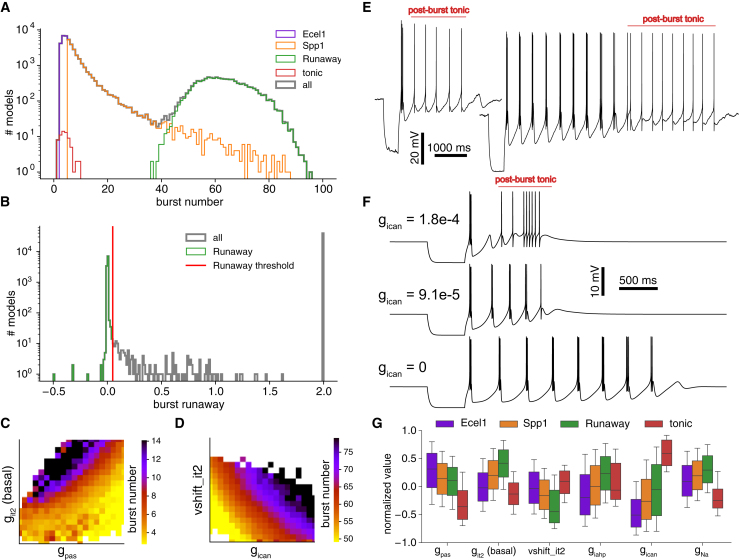
Electrical types analysis with the models (A) Distribution of burst number for the three cell types and cells with tonic firing after rebound bursting, which we refer to as post-burst tonic. (B) Distribution of runaway measure to detect runaway bursting (smaller than 0.05). Values of 2 indicate large runaway values or undetectable ones. (C) Average burst number corner plot for Ecel1/Spp1 models with two most correlated parameters. (D) Average burst number corner plot for Runaway models with two most correlated parameters. (E) Example of experimental traces with transition to post-burst tonic (Ecel1+ left, Spp1+ right). (F) Transition from a model with post-burst tonic firing (top trace) to an Spp1 model by means of decreasing $I_{\text{CAN}}$ conductance (lower traces). (G) Distribution of parameters across cell types, for the statistically significant parameters only, normalized by their bounds.

We began by differentiating electrical cell subtypes based on their electrical bursting properties. Our initial focus was on burst number, (see STAR Methods for calculation), which exhibited a bimodal distribution (Figure 3A, also Figure 2D). Ecel1 models showed a peak at low burst numbers, while Runaway models peaked around 70 bursts. In experimental recordings, with a shorter 14-s duration compared to the 25-s MCMC simulations, the peak corresponding to Runaway cells was centered around lower burst numbers.

To distinguish Ecel1 from Spp1 models, we established a threshold based on a burst number of 5, which corresponded to the experimental observations in the data (see Figure 1D). It is important to note that this classification was not strictly rooted in biological rationale, as there exists a recognized continuum of burst numbers among Ecel1+/Spp1+ cell types. Furthermore, a subset of experimental recordings originated from cells co-expressing Ecel1 and Spp1, positioning them along a transcriptomic gradient that spans the functional range between Ecel1 and Spp1 profiles.^8^ Our sampled models similarly revealed this continuum, ranging from strictly Ecel1-like low-bursting to the Spp1-like high-bursting electrical type.

To further distinguish Runaway models from Spp1 models, a burst count threshold of approximately 40 could be applied. However, this approach would incorrectly classify Spp1 models with high burst frequencies as Runaway cells. We thus defined an additional electrical feature, the burst runaway metric, measuring the slope of the slow AHP depth between bursts (see Figure 3B and STAR Methods for exact feature definition). We observed that traces with a burst runaway metric of less than 0.05mV/ms corresponded to models characterized by bursts that remain stable over time and are unlikely to cease rebound burst firing for an extended duration. This thresholding technique separated the second peak of models in the burst number distribution, exposing a long tail in the burst distribution for Spp1 models that exhibited high-frequency bursts (refer to Figure 3A). We found that Runaway cells generated APs toward the far end of the curve, as illustrated by the time to last spike feature summarized in Figure S5A. Examining this parameter space allowed us to obtain additional insights into the bimodal nature of the burst number distribution.

**Figure 4:**
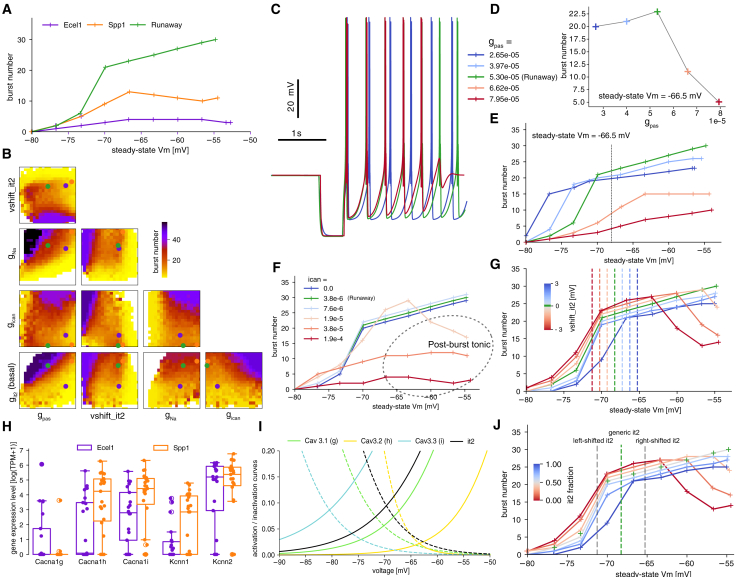
Rebound burst curves (A) Burst number as a function of steady-state voltage for Ecel1, Spp1, and Runaway models. (B) Corner plot of average burst numbers for key parameter pairs. The three models from (A) are marked with dots of corresponding color. (C) Sample traces illustrating the effect of modifying g_pas_ (trace colors correspond to g_pas values outlined in (D). (D) Burst number as a function of $g_{\text{pas}}$ for a fixed steady-state voltage of −65.5mV. (E) Change of $g_{\text{pas}}$ affecting burst number curve of a Runaway model (green). Vertical line represents the curve in (D). (F) Change of $I_{\text{CAN}}$ affecting burst number curve of a Runaway model (green). Traces segments within the dashed oval are ones with tonic firing following the bursts. (G) Change of $I_{T}$ voltage shift affecting the burst curve of a Runaway model (green). Vertical dashed lines represent the center of window current of the generic $I_{T}$ conductance. (H) Cav3 and SK channel subtypes gene expression levels in Ecel1+ and Spp1+ cells (data fromLi et al.^8^). (I) Activation and inactivation curves of Cav3 channel subtypes from McRory et al.^16^ and the generic $I_{T}$ conductance model. (J) The effect of replacing a single $I_{T}$ conductance (green dashed vertical line) with two $I_{T}$ at 3 mV shifted left and right (dashed gray vertical lines), with half the conductance (gray line at it2 fraction of 0.5) is close to the model with a single generic $I_{T}$ channel (green crosses). Relative fractions of each channel have a similar effect to a voltage shift. Notice that the extreme curves blue and red are the same as in (I).

In Figures 3C and 3D, we display the average burst number for a pair of the most highly correlated parameters associated with this feature, for the transitions from Ecel1 to Spp1 and from Spp1 to Runaway models, respectively. Each transition was governed by a unique set of correlated parameters to control this electrical feature. To elaborate further, for low bursting cells, the levels of $I_{T}$ conductance and $g_{\text{pas}}$ played a crucial role in regulating the observed burst count. In contrast, for models firing a higher number of bursts, the conductance of $I_{\text{CAN}}$ and the positioning of the T-type window current (a phenomenon referring to the sustained influx of ${\text{Ca}}^{2+}$ through ${\text{Ca}}^{2+}$-gated ion channels, relying on the overlap between steady-state activation and inactivation^23^) became significant factors. These differences stemmed from the fact that for Runaway models, burst number represents only the burst frequency, while for non-runaway ones it also captures how fast the rebound bursting ends. This qualitative difference suggested that distinct parameter sets modulate burst number in each regime. In Figure 3C, we observe larger burst counts for larger $I_{T}$ conductance in the basal dendrites, where ${\text{Ca}}^{2+}$ influx is stronger and thus more rebound bursts are expected, while for larger passive conductance, the rheobase is also larger, hence ${\text{Ca}}^{2+}$ influx is less likely to generate Na spikes in the soma. In Figure 3D, a larger voltage shift (corresponding to an $I_{T}$ window current centered at more negative voltages), results in a higher frequency of bursts because the rebound bursts are generated at a lower voltage and the membrane potential gets hyperpolarized faster after a burst to trigger the next one, see Cain and Snutch^22^ for a similar discussion. A larger amount of $I_{\text{CAN}}$ conductance also results in a higher number of bursts as $I_{\text{CAN}}$ depolarizes the cell once it is hyperpolarized after a burst, effectively shortening the time between two bursts. These trends were also revisited in Figure 4 in the context of burst curves, and in Figure S5B where we show a prediction of an XGBoost regression model of burst number from model parameters. In these two regimes, we obtained high accuracy (fraction of resp. 0.927±0.002/0.956±0.001 of correct prediction of burst number for resp. low/high groups) only when fitted separately from each other, confirming that the burst number feature captures different underlying mechanisms, depending on the cell type.

Experimentally, it is common to observe tonic spiking following rebound bursting, which we refer to as “post-burst tonic” to differentiate from regular tonic firing under depolarization (see Figure 3E). In our model neurons, only a small fraction displayed tonic firing after the initial rebound busting period. To study this behavior, we designed a specific feature to count the number of such post-burst tonic APs, and classified models with positive values as “post-burst tonic”. This feature was delicate to define and it missed cells with a low number of tonic APs, explaining part of our apparent under-sampling of these models, shown in red in Figure 3A. One of the defining parameters for this behavior was a large value of $I_{\text{CAN}}$ (see Figures S5C and S5D). To assess its role, we picked a model with post-burst tonic firing, as illustrated in Figure 3F (top trace), gradually reduced $I_{\text{CAN}}$ to 0 (bottom trace) and observed the transition from an Ecel1 model with post-burst tonic, to an Spp1 model. Having enough $I_{\text{CAN}}$ was not sufficient for the presence of post-burst tonic firing, it was also necessary to have low $g_{\text{pas}}$ and low $I_{T}$ in basal dendrites, as well as low Na, as seen in Figure 3G displaying the distributions of parameters between each cell type in the models. Along the Ecel1-to-Runaway model gradient, we observed a general increase in $I_{T}$, $I_{\text{AHP}}$, Na, and $I_{\text{CAN}}$ conductances, coupled with larger voltage shifts and lower $g_{\text{pas}}$, validating our previous analysis. The voltage shift differences between cell types resulted from larger shifts reducing burst number, due to fewer open T-type ${\text{Ca}}^{2+}$ channels at −65 mV (see Figure 4H), favoring Ecel1 classification with this −65 mV steady-state protocol.

### Burst curve variability and T-type ${\text{Ca}}^{2+}$ channels

Next we studied the relationship between burst curves and T-type ${\text{Ca}}^{2+}$ channels, particularly burst curve variability induced by the relative fraction of conductances of the Cav3 channel subtypes. First, we show in Figure 4A the burst curves of three electrical models of each subtype, selected from those which attain a maximum burst number below −55 mV. We show the burst number for a simulation time of 14s, as in the experimental data. For this duration of recordings, most data points above 15 bursts were of Runaway type (with a small runaway metric). In the experimental data summarized in Figures 1C–1E, we observe a large variability in the shape of burst curves, in particular in terms of the location and amplitude of its peak. We aimed to determine if our models, which incorporate only the generic T-type ${\text{Ca}}^{2+}$ conductance and omit the specific T-type ${\text{Ca}}^{2+}$ channel isoforms expressed in reticular neurons *in vivo*, could accurately represent these characteristics of the burst curve. In Figure 4B, we present a corner plot of the average number of bursts across pairs of parameters that effectively controlled this feature recorded at steady state voltage of −65 mV.

Although a comprehensive examination of all correlations was not feasible, we conducted a detailed analysis of two specific ones. First, in Figures 4C–4E, we considered the effect that modifying $g_{\text{pas}}$ has on the burst curve. In Figure 4C, we show three traces of bursts at three levels of $g_{\text{pas}}$ (green is the Runaway model, blue and red are lower and higher amount of $g_{\text{pas}}$). The effect of $g_{\text{pas}}$ on burst number exhibited a nonlinear relationship, with a decrease in the number of observed bursts at both high and low values of $g_{\text{pas}}$, as clearly seen in Figure 4D. At high values (orange/red curve), the runaway regime was lost. Conversely, at low values, the cell exhibited runaway rebound bursting over a large range of steady state voltages (all points above 15 bursts are runaway). This is compatible with the previous result presented in Figure 3C, where large $g_{\text{pas}}$ is correlated with a low number of bursts. In Figure 4E, we show multiple burst curves with various $g_{\text{pas}}$ levels, illustrating that a single parameter induces non-trivial variability in the shape of the burst curve. The membrane’s passive conductance, $g_{\text{pas}}$ , reflects the ease with which ions flow across the neuronal membrane through passive channels, thereby influencing membrane potential and its response to inputs. At lower $g_{\text{pas}}$ values (blue/green traces), increasing $g_{\text{pas}}$ accelerates the cell’s dynamics during hyperpolarization (between bursts), resulting in shorter interburst intervals and increased burst numbers (see traces in Figure 4C). However, a sufficiently large increase in $g_{\text{pas}}$ shortens the hyperpolarization period to the point where insufficient ${\text{Ca}}^{2+}$ channels deinactivate, preventing a strong low-threshold spike and subsequent rebound burst (red curves), thus reducing burst numbers. Similarly, altering $I_{\text{CAN}}$ also affects burst dynamics, as previously shown in Figure 3D and further illustrated in Figure 4F. In this case, $I_{\text{CAN}}$ depolarizes the cell, shortening interburst intervals at low values and preventing rebound bursting at high values. While the trends are similar to those observed with $g_{\text{pas}}$ , the mechanisms differ: $g_{\text{pas}}$ speeds up the dynamics, whereas $I_{\text{CAN}}$ drives the membrane potential to higher voltages. At sufficiently high $I_{\text{CAN}}$ values and steady-state voltages, post-burst tonic spiking is observed (dashed circle).

A crucial property of the $I_{T}$ conductance for the burst curve is the location of its window current, or the range of voltages at which there is a significant likelihood of ${\text{Ca}}^{2+}$ influx through the channels, effectively creating a window for current to flow. In Figure 4G we show the impact of varied voltage shift parameter of the $I_{T}$ channel on burst number (base Runaway model shown in green). Apart from an expected shift of the burst curve, the maximum number of bursts (or burst frequency) is also affected (see Figure 3D). For negative voltage shifts, which result in the window current being adjusted to lower voltages, the burst curve also shifts and reaches its peak at these lower voltages. For positive voltage shifts, there is no sharp decrease in burst numbers below −55 mV but an overall reduction of the burst frequency where the runaway type of steady bursting starts at higher steady-state voltages. Relying solely on traces obtained from a single steady-state voltage leads to degenerate models since numerous burst curves can produce the same number of bursts at a given voltage. As a result, constructing a model of a specific cell requires considering the entire burst curve to eliminate this degeneracy. Here, we were rather interested in capturing the variability of the experimental burst curves, hence, we used a set of three steady-state voltages, with large standard deviations of target burst numbers. The observed variability in burst curves among cells of the same type suggested that these cells must possess different relative fractions of conductances to produce these experimental phenotypes. So far, our model only included the generic T-type ${\text{Ca}}^{2+}$ channel conductance, with a voltage shift to account for some of the variability of the burst curve. Voltage shifts of ion channel models are often fixed in agreement with experimental conditions (such as liquid junction potential) at which these channels are characterized. In our models, this voltage shift accounted for the relative conductance of the T-type ${\text{Ca}}^{2+}$ family or Cav3 channel subtypes, as we demonstrate in the following text.

A single-cell transcriptomics analysis of Ecel1+ and Spp1+ neurons reported differences in gene expression related to voltage-gated ion channels known to be important shapers of neuronal rebound burst firing.^8^ Ecel1+ cells exhibited higher expression of the Cacna1g gene, which encodes the low-voltage-activated T-type ${\text{Ca}}^{2+}$ channel subunit *α*-1g. In contrast, Spp1+ cells had increased expression of the Cacna1h and Cacna1i genes, encoding the *α*-1h and *α*-1i isoforms, respectively (summarized in Figure 4H). Direct electrophysiological comparison of *α*-1g, *α*-1h, and *α*-1i under identical recording conditions had identified unique functional characteristics of these three isoforms, suggesting distinct contributions to neuronal physiology.^16^ Examining the window current for each channel isoform illustrated in Figure 4I, we see it peaking at progressively more hyperpolarized voltages for *α*-1h, *α*-1g, *α*-1i. Although all three isoforms overlap in their steady-state activation and inactivation properties, those relative differences in the expression level could produce the observed differences in the burst curve characteristics of Ecel1+ and Spp1+ cells. This relation between the relative fraction of Cav3 channel subtypes and the location of the burst curve is visible in Figure 1E, where the center of the burst curve is at higher voltages for Ecel1+, consistent with the higher expression of Cacna1g gene (Cav3.1 channel subtype) for this cell subpopulation. There were also differences in the expression of the small conductance ${\text{Ca}}^{2+}$-activated K^+^ (SK) channels, which are responsible for the afterhyperpolarization (AHP) phase of rebound burst firing. Spp1+ TRN neurons expressed both SK1 and SK2 channel variants, whereas Ecel1+ neurons predominantly expressed the SK2 subtype^8^ (see Figure S8 for more gene expression comparison between cell types). Due to differences in the activation and inactivation kinetics, as well as ${\text{Ca}}^{2+}$ sensitivity of these SK channel isoforms, TRN neuron subpopulations exhibited varying AHP timescales based on their specific SK channel isoform expression profiles.

As a first approximation, we treated the Cav3 channel subtypes as voltage-shifted versions of the generic $I_{T}$ conductance, disregarding any variations in characteristics like time constants. By substituting the single generic $I_{T}$ with two copies that are shifted up and down by the same voltage, $v_{\text{shift}}$, and halving the overall maximal conductance, we developed a cell model comprising two Cav3 subtypes rather than just one generic channel. For sufficiently small $v_{\text{shift}}$, that is when the window currents of the shifted channels still overlapped significantly, the artificial subtype model was considered similar to the original it2 model. In the limit of vanishing $v_{\text{shift}}$, it would converge to the generic model. In Figure 4J, we conducted this experiment with $v_{\text{shift}}=3\;\text{mV}$ and indeed, the burst curves remained similar. Next, by introducing an imbalance in the conductances of the up and down-shifted $I_{T}$ model, as illustrated in the same panel, we could manipulate the position of the burst curve, similar to how we adjusted the voltage shift of the single channel in Figure 4G. This numerical experiment shows that the voltage shift of the generic $I_{T}$ conductance can be considered an approximation of the relative conductances of the Cav3 channel subtypes. This approach could be used to relate the differential contribution of ion channels to single-cell dynamics in the context of pathologies. For instance, the upregulation of Cav3.2, associated with absence seizures in epilepsy,^24^ would have the primary effect of increasing the amplitude of the burst curve around −65mV (Figure 4I), due to an increase in the magnitude of the overall T-type Ca current around that voltage range (Figure 4A), explaining the hyperexcitability and enhanced burst firing associated with absence seizures. Dysregulated Cav3 channel activity is also associated with spindle and slow-wave impairments in schizophrenia, with a localized reduction in Cav3.3 activity in TRN as one potential cause of observed spindle abnormalities.^25^ Such a targeted reduction of Cav3.3 currents could be tested in Ecel1 or Spp1 model neurons in isolation, to assess its impact on the burst firing properties of different TRN neuron subtypes.

### Model generalizations to biological temperature and morphological population

After having reproduced the biological variability of rebound burst firing in TRN with our models, we turned to the study of their generalization to higher temperatures (from 25° to 34° Celsius) and a population of detailed reconstructions for the same morphological type. Generalizing the models to physiological temperatures allowed us to place them within the thalamoreticular microcircuit of Iavarone et al.,^7^ with morphological variability as described therein, and thereby study the resulting network dynamics presented in the next section.

**Figure 5:**
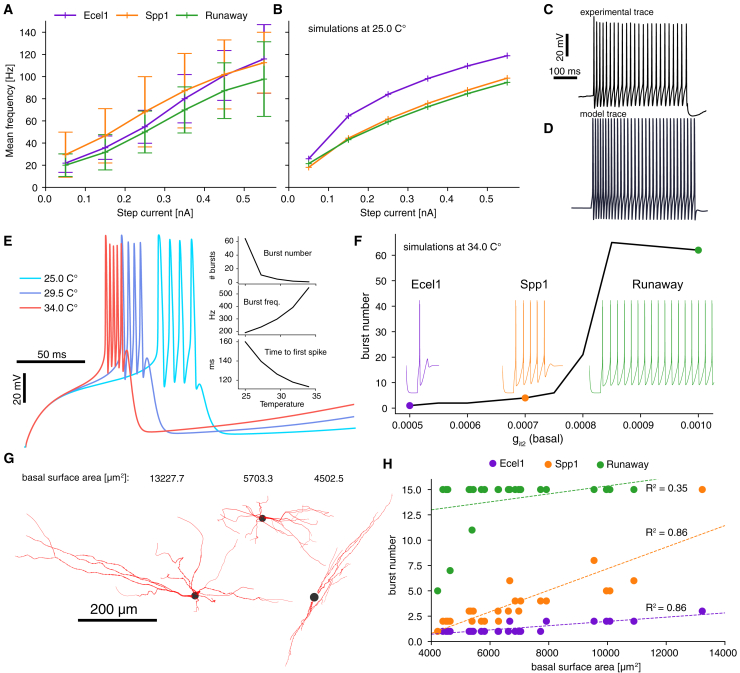
Validation and generalization of electrical models for circuit simulations (A) Experimental IF curves for all three cell types during the tonic protocol at 25°C. (B) IF curve of tonic firing for the three models shown in Figure 2, simulated at 25°C. (C) Representative experimental trace of tonic regime for an Ecel1+ cell under a 0.15 nA step current. (D) Representative model trace of tonic regime for an Ecel1 model cell under a 0.15 nA step current. (E) Traces of bursts when increasing the simulation temperature from 25°C to 34°C. Insets are three features (burst number, burst frequency and time to first spike) as a function of temperature, computed on the full traces (not shown). (F) Number of bursts for a fixed model with the $I_{T}$ conductance in basal dendrites varied. Three highlighted models are ones used as Ecel1/Spp1 and Runaway representatives for network simulations presented later. Traces show rebound bursting of each of the three models. (G) Examples of detailed morphological reconstructions^7^ with various total dendritic surface areas. (H) Burst number versus dendritic surface area for each reconstructed morphology and electrical model. Burst counts are limited to a maximum of 15, and linear regression fits (with R^2^ values) are shown as dashed lines.

In our model development using the MCMC sampling approach, we explicitly excluded any tonic spiking protocols, even though this firing mode plays a crucial role in the network-level activity. Therefore, we validated our models by comparing the current frequency (IF) curve at 25°C with the experimental IF curves. This comparison is illustrated in Figure 5A, where the data have been binned and averaged across all cells of the same type. We did not notice any substantial differences in IF curves across cell types, however, Ecel1+ cells exhibited a greater tendency to enter a state known as depolarization block when subjected to high current injections. We did not attempt to model this phenomenon here, see Hartley et al.^12^ for further details on the experimental data. In Figure 5B, we plot IF curves for the three models at 25° presented in Figure 2, showing that our models are within the experimental range presented in the previous panel. Experimental traces were maintained at −60 mV before the step current injection, so we replicated this condition in the model to ensure consistency. In these three sampled models, we observed that the Ecel1 model had a highest firing frequency. This is explained by our random choice of models (from Figure 2C) that resulted in a near-maximal Na conductance in the soma while the Spp1 and Runaway models had values of Na in the soma near the lower bound (see Figure 2C; Table S2). The IF curve of these three models therefore bounds the range of possible IF curves of the models, in agreement with the variability in the experimental data. Figures 5C and 5D show representative tonic firing traces for both experiments and models, elicited by a 0.15nA current injection. Both exhibited similar action potential shapes and firing frequencies. Our models, constructed without explicit matching of experimental IF curves, nonetheless reproduce them. This suggests that IF curves and neuronal excitability are inherent properties of the burst protocol, rather than additional constraints.

Then, in Figure 5A, we investigated the effect of temperature in our models. Up to now, we constrained the models at 25°C, the temperature used in experimental recordings, but we intended to use them at 34°C in the circuit simulation since this is the physiological temperature. To implement this adjustment, we utilized the q10 temperature coefficient integrated into each ionic mechanism model (see STAR Methods). As illustrated in Figure 5E (insets), the primary impact of raising the temperature from 25°C to 34°C was a reduction in the burst count, accompanied by an increase in both the frequency and the number of action potentials within each burst, along with a decreased time to the first spike. Due to the absence of experimental recordings and their electrical characteristics at 34°C, we relied on the temperature dependence to develop a baseline model capable of replicating the observed burst numbers for each cell type.

Starting from an Spp1 model with a low score (below 2 SDs) at 25°C from the MCMC sampling, we created a baseline Spp1 model at 34°C by increasing the level of $I_{T}$ conductance in its basal dendrites (see also Table S2). This was to compensate for the loss of burst number due to higher temperature (see Figure 5E top inset). Varying the $I_{T}$ conductance in the basal dendrites of this baseline model covered the full range of observed burst numbers of all subtypes (black curve in Figure 5F). We then selected three $I_{T}$ values along this curve to obtain three baseline models, one for each subtype. While simplifying the model by altering only the $I_{T}$ conductance among subtypes was an oversimplification, it allowed for a consistent examination of its impact in circuit simulations by minimizing variability from other parameters.

We then investigated the role of the underlying morphology in determining the validity of each model. In Figure 5G we show three examples of basal dendrites from our dataset of 25 morphologies.^7^ Some morphologies had a smaller dendritic surface area compared to others, yet exhibited a similar level of branching complexity (see Figure S2). In Figure 5H, we present the burst numbers for our three electrical models when assessed across the population of morphologies, plotted against their dendritic surface area. For each cell type, dendritic surface area correlated significantly with burst number, while soma surface area showed a weaker, but still noticeable, correlation. (not shown; slopes (s) and $R^{2}$ values were: Ecel1, s = 0.00087, $R^{2}$ = 0.5; Spp1, s = 0.0037, $R^{2}$ = 0.42; and Runaway, s = 0.00141, $R^{2}$ = 0.17). Therefore, with few exceptions of extremely small or large morphologies, cell types retained their distinct burst number characteristics across the entire morphological dataset. Additionally, the observed correlation in Figure 5B between burst number and $I_{T}$ conductance sensitivity suggested that dendritic $I_{T}$ is more critical for rebound bursting than somatic $I_{T}$ , consistent with prior research.^21^

### The contribution of TRN model neurons to spindle-like oscillations

**Figure 6:**
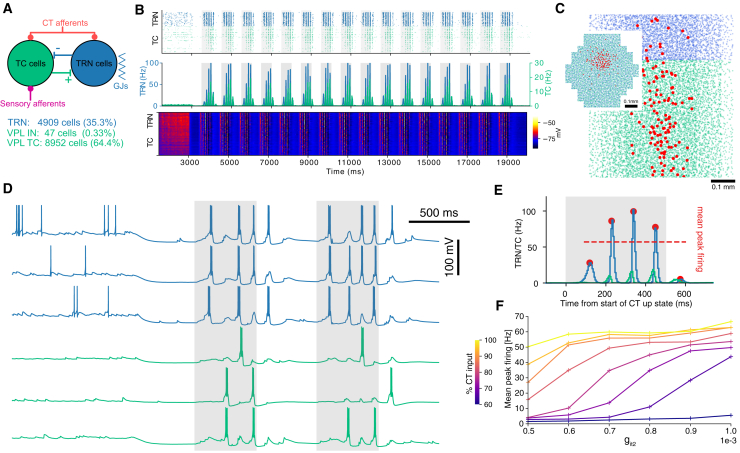
Spindle-like oscillations in response to simulated cortical up and down states (A) Thalamoreticular microcircuit composition and connectivity schematic, including afferent synapses from sensory periphery and cortex as well as gap junction (GJs) connectivity between TRN cells. (B) Population responses spike raster, peri-event time histogram (PETH) and voltage raster (a sample of 500 neurons per type). Gray shadows represent cortical up states. (C) Spatial distribution of cells in the microcircuit column (TRN in blue, TC in green), efferent cells of a single CT afferent are highlighted in red (top view of the column in inset). (D) Example single cell recordings from the simulation in (B). 1,000 ms of wake-like activity followed by first two cortical down and up states. (E) Zoom in on one spindle from (B). Red dots are spindle peaks, dashed line illustrates average mean peak firing. (F) Spindle mean peak firing across various circuits with uniform models in a range of $I_{T}$ conductances, for different fractions of CT inputs.

Thalamic spindle oscillations, commonly observed during cortical up states,^3^^,^^4^^,^^5^ arise from a mechanism involving TRN-mediated inhibition of thalamic relay cells. As demonstrated in the model of Iavarone et al.,^7^ TRN activation leads to TC cell hyperpolarization via inhibitory connections, triggering post-inhibitory rebound and initiating the rhythmic cycle of inhibition and excitation underlying spindles (see STAR Methods for a more detailed account). To delve deeper into the role of specific TRN cell subtypes, we employed the thalamoreticular microcircuit connectome (Figure 6A), preserving synaptic connectivity between all cell populations and gap junction connectivity between reticular cells while replacing original electrical reticular models with varying proportions of Ecel1, Spp1, and Runaway variants to examine their differential impact on spindle propertries.

We have built three circuit instances, composed of Ecel1, Spp1, and/or Runaway model neurons respectively and tested these circuits’ ability to produce spindle-like oscillations in a simulated NREM-like state. Figure 6B presents simulation results for a sample of TRN and TC cells from a circuit instance with all TRN neurons modeled as Ecel1 cells. In this simulation, we observed spindle-like oscillations triggered by simulated cortical up states, reproducing the results of Iavarone et al.^7^ (see Figure 4C). In our study, we extended the sequence of simulated cortical up states to 16 cycles, as a reasonable trade-off between simulation duration and accuracy of averaged quantities such as mean peak firing. This measure was computed as the average firing rate of the peri-event time histograms (PETH) of each spindle event, summarized in Figure 6E. The average and standard deviations of the mean spindle peak firing during a simulation were further used to characterize the ability of a circuit to produce spindle-like activity following the onset of simulated slow oscillations in cortical afferents.

In the model of Iavarone et al.,^7^ all the placed CT synapses present in the circuit were recruited to participate in cortical up states, input which we refer to as 100% CT activation. In this study, we were interested in characterizing how differences in intrinsic cellular excitability of TRN subpopulations contribute to emergent spindle dynamics. To this end, we varied the strength of synaptic depolarization by recruiting different percentages of CT afferents during each up state, starting from 40% up to 100%. Figure 6F presents the mean peak firing levels for several intensities of CT synaptic inputs and a range of dendritic $I_{T}$ conductances. The electrical models depicted in Figure 5F were used, with only the dendritic $I_{T}$ conductance modified to span the full range of the three cell types, from an Ecel1 model to Runaway models with maximal $I_{T}$. For weak CT activation below 60%−70%, peak firing levels were minimal, indicating an inability of the circuit to reliably generate spindle-like oscillations at this level of synaptically driven depolarization. Stronger CT activation resulted in more pronounced peak firing levels, particularly in circuits with higher $I_{T}$ conductance levels. At 100% CT input, all circuits exhibited similar peak firing levels above 40 Hz, a threshold indicative of large spindle-like oscillations. This result demonstrated a strong correlation between the amount of cortical synaptic input received by the thalamus, the overall ${\text{Ca}}^{2+}$ currents in TRN cells and the ability of the circuit to generate spindles.

### $I_{T}$ and $I_{\text{AHP}}$ conductances predict the mean peak firing rate of spindle-like oscillations

The TRN is segregated into modality-specific sectors based on its innervation of particular thalamic nuclei and reciprocal connections with specific cortical areas.^1^ This anatomical organization results in regionally specific oscillatory properties, which could account for differences in local sleep-wave activity. It has been demonstrated that oscillatory burst firing varies across TRN sectors, with sensory sectors exhibiting more repetitive burst firing compared to the limbic sector.^26^ Hence, one critical aspect we addressed with this modeling work was the impact of TRN circuit composition in terms of Ecel1/Spp1 ratios on spindle dynamics. Since precise cell-type proportions for individual sensory sectors, such as the somatosensory region, were not available, we relied on the findings of Hartley et al.^12^ and Li et al.^8^ to establish a baseline composition for our model. Li and colleagues^8^ reported approximately equal fractions of Ecel1+ and Spp1+ cells within the whole TRN (Figures 2C and 3C). Further investigation revealed that roughly 50% of the recorded Spp1+ cells exhibited a runaway bursting phenotype (Hartley et al.,^12^ Figure 2N). Based on this, and building on the previous section’s uniform TRN circuits, we systematically varied cell-type fractions to create mixed circuits with varying cell compositions to explore the relationship between effective $I_{T}$ conductance and mean peak firing during spindle-like oscillations.

**Figure 7:**
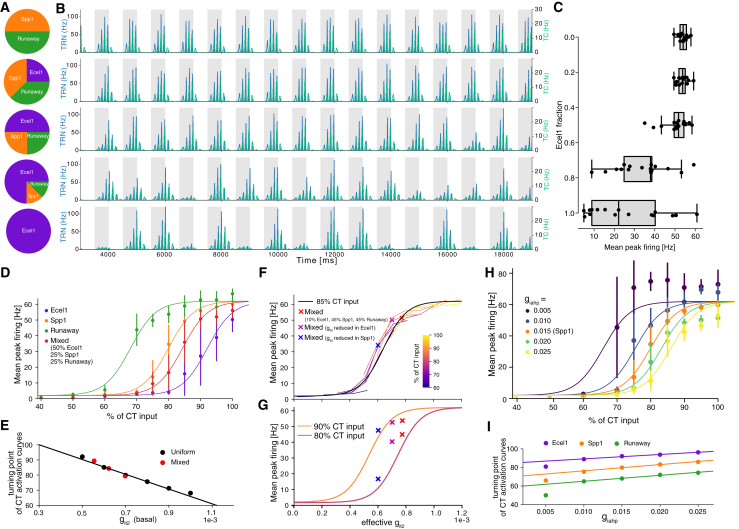
Peak firing of spindle-like oscillations can be predicted from circuit composition (A) Illustration of circuits with different cell compositions, varying the fraction of Ecel1 cells. (B) TRN and TC spikes PETH for each of the circuit variants in (A) during cortical up/down states (highlighted in gray). (C) Spindle peak firing as a function of the fraction of Ecel1 cells in the circuits introduced in (A). Each dot represents the mean peak firing during one CT cycle (up and down states, 16 per simulation). (D) Spindle peak firing as a function of CT input for three uniform circuits with the three base models, and a circuit with Ecel1 fraction of 0.5 from (A). We fitted only the position of the sigmoid function (CT turning point), while all other parameters were kept the same. (E) Sigmoid centers from (D), or CT turning points as a function of effective circuit $I_{T}$ conductance (defined as a weighted harmonic mean of a model’s basal dendrites $I_{T}$ conductance). The black line is a linear fit. The mixed circuits are the three middle variants from (A). (F) Mean peak firing at 85% CT input (black sigmoid) from the linear fit in (E) (black line) as a function of $I_{T}$ . Colored curves are those depicted in Figure 6H, shifted along the *x* axis by using the slope of (E). Crosses indicate the mean peak firing at 85% CT input in three circuit configurations: 10% Ecel1, 45% Spp1, and 45% Runaway models (red); the same circuit with reduced $I_{T}$ in Ecel1 models (magenta); and the same circuit with reduced $I_{T}$ in Spp1 and Runaway models (blue), reproducing the experiment of Li et al.,^8^Figure 5. (G) Similar to (F), but for 90% and 80% CT input. (H) Spindle peak firing as a function of CT activation levels for circuits with various levels of $I_{\text{AHP}}$ conductance. (I) Same as (E) but with varying $I_{\text{AHP}}$ conductances for each of the three base models (with $I_{\text{AHP}}$=0.015). Linear fit ignores the points at 0.005 as bursts are not clearly defined in the simulation.

Figures 7A–7C show circuit activity with increasing Ecel1 fractions at 90% CT input. Higher Ecel1 fractions, with lower total $I_{T}$ conductance, lead to decreased peak firing. While peak firing decreased, spindles still occurred, albeit less reliably, as seen in the increasing variability with increasing fractions of Ecel1 cells in the circuit (Figure 7C).

To gain deeper insight into the relationship between the level of $I_{T}$ conductance in the basal dendrites and spindle-like oscillations, we plotted in Figure 7D mean peak firing levels of spindles as a function of CT synaptic input for three uniform circuits and one mixed circuit variant. For each of these circuits, the dependence of spindle mean peak firing on CT input exhibits a sigmoidal relationship, with the primary variation occurring in the location of the turning point influenced by the level of $I_{T}$ conductance in the respective model neurons. To quantify this relationship, we fitted sigmoid functions to the data of several uniform circuit models (black dots in Figure 7E), fixing all parameters except for the turning point location. The resulting turning point values were then plotted against the corresponding dendritic $I_{T}$ conductance levels in Figure 7E. This relation was linear, with high confidence, suggesting that the amount of $I_{T}$ currents in basal dendrites was predictive of the mean peak firing observed during spindle activity in these circuits. To further analyze the mixed circuits depicted in Figures 7A and 7B, we applied the same sigmoid fitting procedure and computed the effective $I_{T}$ conductance for each cell model. This effective $I_{T}$ conductance was calculated as the weighted harmonic mean of the dendritic $I_{T}$ conductances of the individual models, weighted by their relative fractions within the circuit. The use of the harmonic mean was appropriate given the density-like nature of conductances. As illustrated in Figure 7E (red dots), effective $I_{T}$ conductance emerged as a reliable predictor of the mean peak firing during spindle-like oscillations, following the same linear trend as was observed for circuits composed of uniform cell models (black dots). This finding suggested that the level of peak firing during spindle activity could be inferred from the effective $I_{T}$ conductance in basal dendrites across various circuit compositions. The linear dependence between the sigmoid turning point and the level of $I_{T}$ conductance (see Figure 7E) allowed us to establish a mapping between cortical synaptic input and effective $I_{T}$ conductance for maintaining a constant spindle mean peak firing frequency. As a further validation, we did a parameter change using this mapping to represent the $I_{T}$ scans of Figure 6F as a function of the effective $I_{T}$ conductance at 85% of CT input (colored lines), which superimposed onto the sigmoid model (black line), as depicted in Figure 7F. This predictive power of the effective $I_{T}$ conductance enabled us to constrain the cell compositions of a circuit from its mean spindle peak firing, or rather spindle robustness or reliability. For example, it was found that a reduction of ${\text{Ca}}^{2+}$ currents in Ecel1+ cells has only a marginal effect on the average number of spindles, but a similar reduction of ${\text{Ca}}^{2+}$ currents in Spp1+ cells induces a significant decrease in the number of spindles.^8^ In Figures 7F and 7G, we show the result of a similar experiment in our model. We built a circuit with 10% Ecel1 cells (red cross), then reduced $I_{T}$ conductance by 0.0002 independently in either Ecel1 models (magenta cross) or Spp1/runaway models (blue cross) to create two additional circuit variants. Only the circuit with the reduction in Spp1 cells had an effective reduction of $I_{T}$ large enough to decrease mean spindle peak firing, similar to Li et al.,^8^Figure 5. Hence, this modeled correlation between $I_{T}$ conductances and spindle peak firing may serve as an implicit constraint on TRN sector cell composition from experimental data. In this case, the constraint was on the maximal fraction of Ecel1 cells of about 10%−15% such that reducing $I_{T}$ conductances in Spp1 cells produced a larger drop in effective $I_{T}$ than the reduction in Ecel1, and reproduced the result of Li et al.^8^ While circuit composition dictated the location of the circuit along the black sigmoid curve in Figure 7F, the percentage of CT afferents recruited during each up state controlled the location of the sigmoid curve along the *x* axis. As shown in Figure 7G with 80% and 90% of CT inputs engaged, the mean peak firing frequencies of the mixed circuits still followed the sigmoid curve, but with stronger synaptic depolarization, the reduction of $I_{T}$ in Spp1 cells did not produce a sharp decrease in mean peak firing any longer. Thus, the percentage of CT afferents recruited during cortical up states, which is difficult to constrain experimentally, was also important for this prediction.

Next, we characterized the effect of varying the conductance of SK channels, responsible for the AHP current, on mean peak firing. When activated, SK channels hyperpolarize reticular cells, enabling them to repeatedly rebound burst fire while regulating both burst frequency and regularity.^27^ In the TRN, downregulation of SK channels has been shown to increase burst duration and frequency, whereas upregulation increases AHP amplitude and reduces intrinsic excitability, as demonstrated by Silvan and colleagues^28^ and also Figure S7. In Figures 7H and 7I, we present a similar analysis of the observed mean peak firing while varying the level of $I_{\text{AHP}}$ conductances in basal dendrites. We observed similar sigmoid profiles, although with an opposite trend. For a fixed level of CT synaptic input, increasing the level of $I_{\text{AHP}}$ conductance resulted in a decrease in peak firing. At the lowest level of $I_{\text{AHP}}$ conductance (at 0.005), the circuit did not exhibit regular and well-defined spindle-like oscillations. Instead, the neurons were bursting for most of the simulation duration, irrespective of the level of cortical inputs. This behavior could be attributed to the shorter hyperpolarization periods associated with low $I_{\text{AHP}}$ levels, which allowed the cells to trigger subsequent bursts more rapidly. As the level of $I_{\text{AHP}}$ conductance increased, each burst comprised fewer action potentials, and the subsequent hyperpolarization period became longer, making it less likely for the the cell to burst on subsequent cycles of the spindle (Figure F). This resulted in reduced mean peak firing for the same level of CT input (see Figures 7H and 7I). In Figure 7I we performed a linear fit (excluding the lowest $I_{\text{AHP}}$ point at 0.005) for the CT turning point of $I_{\text{AHP}}$ scan of Figure 7H, similar to the fit in Figure 7E. We conducted the same analysis for the other two uniform circuits, composed of either Ecel1 or Runaway cells, and observed a similar positive slope, independent of their value in $I_{T}$ . This demonstrates that $I_{T}$ and $I_{\text{AHP}}$ conductance levels have opposite but uncorrelated effects on mean spindle peak firing and can be used in concert to predict the mean peak firing in a circuit during spindle-like activity.

## Discussion

In Li et al.,^8^ and more recently in Hartley et al.,^12^ neurons in the thalamic reticular nucleus have been experimentally studied in the context of understanding how intrinsic cellular properties impact emergent network-level dynamics. It was found that reticular neurons can be segregated into two transcriptionally defined populations, Ecel1+ and Spp1+, named after two genes most differentially expressed by these neuronal subpopulations. Based on these experimental results, we tasked ourselves with creating detailed numerical models of these cell types and investigating their properties, from ion channels to circuit dynamics with spindle-like oscillations. Leveraging the MCMC sampling method for building detailed electrical neuron models,^15^ we obtained a large pool of TRN models matching the experimental variability of electrophysiological features across the two cell types. Consistent with the findings of Li and colleagues,^8^ we did not observe a clear-cut distinction between Ecel1+ and Spp1+ TRN cell subpopulations, but rather a continuum of models with smooth transition in properties, such as maximal rebound burst firing. Furthermore, the electrophysiology dataset underlying our modeling effort contained a significant proportion of cells that exhibited prolonged bursting activity, referred to as runaway neurons.^12^ We were able to generate models of these cells by sampling from the same MCMC parameter space, and by exploring it, we identified the ion channels that primarily control the transitions between different cell subtypes. While T-type ${\text{Ca}}^{2+}$ and SK ($I_{\text{AHP}}$ ) channels were the most important ion channels in shaping the bursting behavior of TRN neurons, the balance between active and passive membrane properties, such as the ratio of passive to active conductances ($g_{\text{pas}}$/ $I_{\text{CAN}}$ or $g_{\text{pas}}$/ $g_{\text{Na}}$ ), also played a crucial role. These passive properties influenced the overall excitability of the neuron and, consequently, its ability to generate bursts.

Due to a lack of complete and coherent ion channel models that account for the different isoforms of T-type ${\text{Ca}}^{2+}$ and SK channels, we were constrained to using generic models. To address this limitation, we considered a strategy involving duplicated generic $I_{T}$ channels, which were symmetrically shifted in voltage. We demonstrated that the relative conductances of these duplicated channels correspond to a voltage shift of a single generic $I_{T}$ channel. This result indicates that, as a first approximation, the diverse properties of different channel subtypes can be modeled by adjusting the density and voltage sensitivity of a generic channel model. By manipulating these parameters, we could effectively simulate the behavior of different channel isoforms. This simplified approach proved particularly useful in capturing a significant portion of the variability observed in the burst curves, which quantified a cell’s ability to rebound burst when subjected to hyperpolarizing current injections.

Building on the work of Iavarone et al.,^7^ where a microcircuit of the somatosensory thalamus was built and validated to subserve spindle-like oscillations, and retaining the original connectome, we have incorporated Ecel1 and Spp1 electrical models into the TRN tier of the circuit and explored the impact of intrinsic ionic conductances on network activity. We observed that both $I_{T}$ and $I_{\text{AHP}}$ conductances of TRN cells were accurate predictors of mean peak firing during spindle-like oscillations. Consistent with experimental evidence,^27^ our model showed that these conductances exerted opposing influences on spindle peak frequency, demonstrating its ability to reproduce the physiological mechanisms underlying stable bursting. When considering circuits composed of diverse cell subtypes (mixed circuits), an effective $I_{T}$ conductance (defined as the harmonic weighted mean of individual $I_{T}$ conductances) proved to be a reliable predictor of spindle peak firing. We propose that manipulating the $I_{\text{AHP}}$ conductance in these mixed circuit configurations would yield a comparable, yet opposite, relationship. The linear model with effective $I_{T}$ conductance allowed us to reproduce the knock-out experimental result of Li et al.^8^ Figure 5, whereby the reduction of ${\text{Ca}}^{2+}$ currents in Ecel1+ cells had a non-significant effect on spindle oscillations but a similar modification in the Spp1+ subpopulation visibly reduced spindle numbers, spindle duration, and led to fragmented sleep. Reproducing this experimental result provided bounds on the cell composition of the circuit, and in particular on the maximum number of Ecel1 cells as compared to Spp1 cells of around 10%−15% such that this effect could be observed. This bound in cell composition cannot be directly compared with experimental data but may serve as a basis for predicting the cellular composition of different TRN tiers based on localized sleep-related patterns in modality-specific thalamocortical loops.^26^

Our approach to TRN single-cell modeling diverges from previous studies in several key aspects. Prior models often focused on replicating specific firing properties, with parameters manually adjusted to achieve desired outcomes,^29^^,^^30^ or relied on a single electrical model fitted to mean recording values.^18^ Constrained by the available experimental data and a direct optimization approach,^18^ classified TRN neurons into two electrical types based on tonic discharge, resulting in models that were only capable of generating one or two bursts.^7^ We extended this by generating a population of biophysically detailed models that capture the observed heterogeneity and continuum of electrical properties within the TRN, moving beyond the limitation of single representative models for each cell type. While simplified models, such as those recently developed by Wang et al.,^31^ may be relevant for lighter and faster circuit simulations, providing insights about qualitative changes in the system’s behavior, biophysically detailed models accurately capturing biological variability observed experimentally are needed for pinpointing the fundamental mechanisms of various thalamic functions.

Overall, our numerical model provides a platform for understanding the role of TRN neurons in sleep rhythms and its dysfunction in disorders, such as epilepsy and schizophrenia, enabling testable predictions. Specifically, this model can be used to elucidate the complex and debated role of TRN neurons in absence epilepsy, a thalamocortical network disorder characterized by spike-and-wave discharges, the electrophysiological hallmark of absence seizures. While it is well-established that aberrant burst output of TRN neurons is implicated in the pathology of absence epilepsy, the precise mechanisms remain a subject of considerable debate. For instance, Cain et al.^24^ suggest that increased activity of TRN Cav channels, particularly Cav3.2, drives abnormal rebound bursting, thus promoting seizure propagation.^24^ Conversely, Abdelaal et al.^32^ propose that TRN dysfunction, characterized by diminished rebound bursting and reduced T-type Cav currents, can itself induce absence seizure-like activity.^32^ Further complicating the picture, McCafferty et al.^33^ demonstrate that only a subset of TRN neurons exhibit increased burst firing during experimental absences in rats, and that a reduction in overall TRN bursting by means of blocking Cav channels is primarily responsible for reduced seizure incidence. These discrepancies underscore the complexity of thalamocortical circuitry in absence epilepsy and likely arise from variations in experimental models, manipulations, and the specific neuronal subpopulations examined. The refined thalamoreticular circuit model we present here captures the diversity of TRN neurons observed experimentally, includes the dynamics of relevant ion channels as well as detailed synaptic connectivity between TRN and TC cells. This enhanced model offers the potential to systematically test the contribution of different scenarios of TRN dysfunction and its underlying mechanisms to seizure initiation, propagation, and termination—manipulations that are all technically challenging to perform *in vivo*.

In addition to its relevance for epilepsy, several sleep studies report marked deficits in sleep spindles as well as slow-wave abnormalities in schizophrenia.^25^^,^^34^^,^^35^ From a translational perspective, developmental disruptions in the expression of risk genes, particularly those highly expressed in the TRN and associated with disorders like schizophrenia (e.g., Cav3.3), could potentially alter the balance of Ecel1-like and Spp1-like neurons, impacting spindle generation through broader transcriptional changes. This raises important questions about the influence of ion channel mutations, such as those in Cav3.3, on the developmental trajectory of these neuronal subtypes. While Cav3.3 loss of function might shift neuronal phenotypes toward an “Ecel1-like” bursting mode, it could also significantly impact cell composition and the overall architecture of thalamic circuitry. Interestingly, our model revealed that as the proportion of Ecel1 neurons increased within the circuit, intermediate-strength CT up states failed to consistently trigger spindles on each up state. This instability in spindle generation, particularly in these conditions, can be investigated in detail using our numerical model to elucidate the underlying mechanisms. Furthermore, impaired TRN activity has been linked to schizophrenia-related abnormalities in cortical activity during wakefulness, particularly in the delta and gamma bands.^25^ This connection is reinforced by Hartley and colleagues,^12^ who showed that inhibiting specific TRN subnetworks produced distinct cortical activity abnormalities, including spontaneous or evoked gamma oscillations. Our enhanced thalamoreticular microcircuit, validated for realistic spontaneous and evoked wakefulness activity,^7^ is uniquely positioned to probe the contribution of cellular level dysfunction to network level pathological oscillations. By developing and integrating biophysically detailed reticular models that capture experimental heterogeneity into network simulations, our work provides a robust foundation for future research. This multi-scale approach enables a more systematic investigation into how the diversity of reticular cell properties dictates thalamic network dynamics and function in both physiological and pathological states.

### Limitations of the study

This modeling work faces several limitations. Primarily, our ability to precisely capture isoform-specific properties, particularly in reproducing highly skewed burst curves, was limited by the lack of complete and coherent ion channel models for different isoforms of T-type Ca^2+^ and SK channels, necessitating the use of generic models. Furthermore, other kinetic factors, such as variations in time constants, may be crucial for accurately modeling these complex firing patterns.

Regarding our modeling approach, although the MCMC-driven method produced a large ensemble of electrical models capturing key aspects, deviations between model and data arose from our reliance on a limited range of steady-state voltages, rather than the full experimental burst curve. Future research should explore incorporating the entire burst curve to improve model accuracy.

Another constraint was the simplified representation of dendritic conductance. While we incorporated a linear increase in the density of the IT conductance in the basal dendrites, we did not conduct a detailed analysis of the specific impact of this distribution on the electrical models. Integrating experimental data from dendritic recordings^21^ could help address this limitation and assess the impact of non-uniform conductance distributions on cellular electrical properties. Such an approach could be further linked to morphological properties of cellular subtypes, as some studies report different ratios of surface areas between somatic and dendritic compartments.^36^

Moreover, we did not attempt to reproduce the depolarization block state of Ecel1+ cells, which was frequently observed at high current amplitudes during tonic firing in the experimental data (Hartley et al., 2024). Given that the implications of this depolarization block for circuit-level function are not well characterized, we leave this aspect for future investigations, which will likely require more detailed models of ${\text{Ca}}^{2+}$/SK and Na+ channels.

Additionally, the adaptation of data from 25 to 34°C, while practical, introduced assumptions that warrant further experimental validation. Finally, the findings presented here are specific to the somatosensory TRN and may not generalize to other sectors.

While MCMC sampling yielded a diverse population of single-cell models suitable for examining the full spectrum of TRN cell intrinsic properties, our analysis of spindle properties focused on a limited set of electrical subtypes to establish clear ion channel relationships. Although each neuron model became unique when paired with a morphology from detailed reconstructions (see Iavarone et al.^7^ for details), fully capturing TRN network complexity requires incorporating a broader diversity of single-cell models, as recommended by Arnaudon and colleagues.^15^ This approach would allow for further exploration of how individual cell-to-cell variability impacts network-level behavior not only for sleep rhythms and spindles but also for broader thalamic activity, impacting sensory processing and thalamocortical communication.

## Resource availability

### Lead contact

Further information and requests for resources and code should be directed to and will be fulfilled by the lead contact, Sean Hill (sean.hill@epfl.ch).

### Materials availability

This study did not generate new unique reagents.

### Data and code availability

• All data reported in this paper has been deposited on Zenodo: https://zenodo.org/records/15055541.
• All original code used in this paper has been deposited on Zenodo: https://zenodo.org/records/15055541.
• Any additional information required to reuse the models of this study is available from the lead contact upon request.

## Acknowledgments

This study was supported by funding to the Blue Brain Project, a research center of the 10.13039/501100001703École Polytechnique Fédérale de Lausanne, from the Swiss government’s ETH Board of the Swiss Federal Institutes of Technology.

## Author contributions

Conceptualization, P.L. and S.L.H.; experimental data acquisition, N.D.H. and R.K.; methodology, P.L., and A.A.; software, P.L. and A.A.; validation, P.L., A.A., and S.L.H.; formal analysis, P.L. and A.A.; investigation, P.L. and A.A.; data curation, P.L.; writing – original draft, P.L. and A.A.; writing – review and editing, P.L., A.A., N.D.H., Z.F., and S.L.H.; visualization, P.L. and A.A.; supervision, A.A., S.L.H., Z.F., and G.F.; funding acquisition, S.L.H.

## Declaration of interests

The authors declare no competing interests.

## STAR★Methods

### Key resources table

**Table:** 

REAGENT or RESOURCE	SOURCE	IDENTIFIER
**Deposited data**		

Data and script to reproduce figures	This paper	https://doi.org/10.1101/2024.12.08.627399
Morphological data	Elisabetta Iavarone et al.^7^	https://doi.org/10.5281/zenodo.7562911
Electrophysiological data	Hartley et al.^12^	https://doi.org/10.1101/2024.12.08.627399

**Software and algorithms**		

emodel-generalization	Arnaudon et al.^15^	https://doi.org/10.5281/zenodo.8269363
BluePyOpt	Van Geit et al.^19^	https://doi.org/10.5281/zenodo.8136124
eFEL	https://github.com/BlueBrain/eFEL	
NeuroM	https://github.com/BlueBrain/NeuroM	
Neurodamus	https://github.com/BlueBrain/Neurodamus	
BluePySnap	https://github.com/BlueBrain/snap	
NEURON	https://www.neuron.yale.edu	
Dask	https://www.dask.org	

### Method details

#### Electrophysiologcal data

The experimental protocols to collect *ex vivo* electrophysiology data is described in detail in.^12^ In short, genetically identified Spp1+ and Ecel1+ TRN neurons were subjected to 500 ms long hyperpolarizing current injections at various holding potentials ranging from −80 to −50 mV, with the negative current adjusted to hyperpolarize each cell to a range of −110 to −100 mV.

#### Morphological reconstructions

The morphological reconstructions for both single cell modeling and circuit simulation were taken from.^7^ For TRN cells, the dataset consisted of 25 detailed reconstructions cloned into 7315 morphologies for building the microcircuit. These morphologies were reconstructed and processed by,^7^ including unraveling and repair procedures to account for the various biases induced by the reconstruction techniques. For MCMC sampling of our electrical models, we required a representative morphology. Following,^15^ we used an average morphological model derived from detailed reconstructions, as shown in Figure S2. We first created a model for the soma with a single cylinder of average radius and length such that the surface area matched the average from the population (see Figures S2A and S2B). We then estimated the average surface area density of the basal dendrites as a function of the path distance to the soma from all the reconstructions and selected the morphology closest to this average value (green in Figures S2D and 2B). We noticed that soma and dendritic surface areas were correlated in this dataset ($R^{2}=0.6$), with some variability in the areas, number of neurites and maximum branch orders, see Figure S2E.

#### Burst curves fitting

The burst number of each trace was computed using the feature described below and the steady-state voltage was estimated as the average voltage during the first step of the bursting protocol. We fitted this data with a skewed normal distribution

(Equation 1) $$f\left(\nu \right)=ae^{-\frac{{\left(\nu -c\right)}^{2}}{w}}\left(1+\mathrm{erf}\left(s\left(x-c\right)\right)\right)+d\text{,}$$

with skewness parametrized by *s*, width by *w*, amplitude by *a*, center by *c* and linear shift by *d*. We used the following bounds for these parameters in the fitting function (Scipy curve _ fit function): a∈[0,100], w∈[20,180], c∈[−100,−40], s∈[−2,2] and d∈[0,1].

#### Classification of cell types

To identify the most significant electrophysiological features for cell type classification, we applied an XGBoost classifier with SHAP feature importance analysis^37^ to electrical features extracted from recordings obtained with rebound burst protocols. Doing a 10-fold cross-validation (repeated 10 times) of traces with maximal burst number per cell using the entire set of electrical features extracted from rebound burst protocol data yielded an accuracy of 88.2+−13.7%. Excluding the most obvious differentiator, maximum burst number per holding membrane voltage, and reclassifying the traces using the rest of the electrical features still produced similar accuracy results (86.0+−15.7%). Finally, using only the top three features identified as most influential, namely burst number, burst mean frequency and spike width allowed us to classify the traces with an accuracy of 87.6+−14.3%. The top features in each classification with their Shapely values are shown in Figure S3.

#### Ion channel models

Detailed electrical models were constructed by assigning the following mechanisms to the somatic and basal compartments.

(1) hh2_Na and hh2_K from^13^ (soma). Fast sodium and potassium channels, ena=20mV and ek=−80mV were fixed to approximately match AHP depth and AP amplitude from data.
(2) cad from^13^ (soma and basal): calcium dynamics with linear decay term, decay rate fixed to 150 ms for both compartments to match the shape of slow AHP.
(3) $I_{\text{CAN}}$ from^13^ (basal): calcium-activated nonselective cation, responsible for slow depolarization and the transition to post-burst tonic firing.
(4) $I_{T}$ from^13^ (soma, basal with increasing density away from soma): generic T-type calcium channel that creates low threshold calcium spikes, with an additional parameter for the slope of linear increase away from soma. A voltage shift parameter was included to shift the center of its window current, where positive values shifted toward negative voltages.
(5) $I_{\text{AHP}}$ from^13^ (soma and basal): SK-type calcium activated potassium channel, responsible for the slow hyperpolarisation after bursts. A voltage shift of 10 mV was applied to the reversal potential to match the depth of slow AHP from data.
(6) $I_{A}$ from^38^ (basal): A-type transient potassium channel, important for the timing, frequency, and overall regulation of rebound bursting.
(7) g_pas (all): passive channels with e_pas=−70 mV.

For $I_{T}$ , the increasing density function used was defined as:

(Equation 2) $$g_{\max,it2}\left(d\right)=g_{0,it2}\left(1+\frac{d}{it2_{slope}}\right)\text{,}$$

where *d* is the path distance from the soma.

#### Protocols and features

The protocol used to characterize rebound bursting was kept as close as possible to the experimental one. First, for a period of 5 s, we injected a current to maintain the cell at the specific steady-state voltage. This current was previously found by a bisection search based on a step protocol of 2 s in duration. With the same bisection search, we found the current to hold the cell at −100mV and used it for a 500ms hyperpolarisation step. After this step, we kept the initial holding current for additional 25 s. For MCMC sampling, we used three steady-state voltages, −80mV to ensure no bursts were triggered, −65mV as the main protocol around the peak of the burst curve, and −55mV to prob the burst curve around the largest possible voltage.

From these protocols, we computed the following features: the burst number (see below for details on its computation), number of spikes per burst, burst mean frequency (frequency of spikes in each burst, averaged over all bursts), peak voltage (absolute voltage of the peak), AP2 AP1 peak difference (the drop of spike amplitude between the first two spikes), time to first and last spike, first ISI (inter-spike interval, other ISIs are highly correlated with this one), AHP depth (in absolute voltage, averaged across all spikes), postburst minimum values (or slow AHP depth between bursts, averaged across all bursts), voltage std (std of voltage trace to ensure it is stable), post-burst tonic (number of tonic spikes after the bursting period, see definition below). In Table S1 we show the mean and std of these features that were used for MCMC sampling, set by hand, close to the experimental values.

The depolarizing protocol, not used for modeling, but rather validation of tonic firing properties of the models (Figures 5C and 5D), was similar to the experimental one, where neurons were maintained with a current injection to target a steady state voltage of −60 mV and then subjected to a 500 ms depolarizing current injection of 0.15 nA to monitor tonic discharge.

#### Burst number feature

The burst number was capturing the number of bursts occurring during a trace. The definition of a burst from a trace is not trivial, especially for edge cases, such as for traces with a single burst, or bursts of single a action potential. We tried to design a feature robust to these edge cases as follows, see Figure S4 for an illustration. We computed the inter-spike intervals between all APs of the trace and looked for a clear bimodal distribution, distinguishing the inter- and intra-burst ISIs. The number of inter-burst ISIs plus one gave the number of bursts. For edge cases, e.g., if all ISIs were smaller than 50 ms, we assumed it to be a single burst. If only a few bursts had more than one spike, it was sufficient to detect the bimodal distribution, hence all the subsequent bursts of a single spike could be counted as bursts. If all the bursts were composed of single APs, it was not considered as bursting (a rare edge case).

#### Burst runaway feature

To detect if a cell is of type burst runaway, we used the time to last spike feature, but also a measure of the slope of voltage drift of slow AHP depth between bursts, see Figure S4 for an illustration. We computed the slow AHP depth between each burst (discarding any possible minima before 5 ms after the last AP in a burst), and computed the slope of the voltage drift between the second and the one before the last slow AHP depth. We discarded the first and the last burst, to prevent biased from some unusual start of bursting, cut traces, or unusual transitions to the end of the bursting period. If the drift was present, assumed to be if the slope was larger than 0.05mv/ms, it indicated that the bursting behavior was not sustainable, and would eventually come to a stop at some point (possibly past the end of the recording time).

#### Post-burst tonic number feature

Some traces stop bursting, but still fire action potential in a more regular, tonic manner. We have designed a feature able to detect these spikes not as bursts, but as post-burst tonic spikes, see Figure S4 for an illustration. This feature was also used in the computation of the burst number, to ensure consistency. The number of post-burst tonic APs in a trace was detected as follows: In the bursting regime, the slow AHP depth between bursts was always lower than the fast AHP depth between APs, while for the post-burst tonic regime, they were of comparable depth. Hence, we counted the number of detected inter-burst ISIs which had slow AHP higher than fast AHP depth. This gave us a good estimate of the number of post-burst tonic spikes in a trace (adding one to the number of tonic ISIs).

#### Thalamoreticular microcircuit modeling

The thalamoreticular microcircuit from^7^ was replicated for all network simulations, with the exception of TRN electrical models. For completeness, we provide a concise overview of the circuit’s essential features and refer readers to the original paper for in-depth information.

The microcircuit was defined to span parts of the TRN and VPL of the thalamus. The dimensions of the microcircuit were determined based on the extent of TRN dendrites and anatomical parcellation from the Allen Brain Atlas. The resulting model microcircuit contained approximately 14k neurons composed of TRN, VPL TC and VPL interneuron cells (Figure 6A). Soma positions were determined using a pseudorandom algorithm considering morphological constraints, while synaptic connections were established by presynaptic axons and postsynaptic dendrites/somata. Afferent synapses in the thalamus from the sensory periphery (medial lemniscus) and from the cortex were included using volumetric bouton densities as constraints. The physiology of synapses was modeled using data on short-term plasticity, postsynaptic potential amplitudes, time constants, and reversal potentials. Synapse models featuring stochastic transmission and short-term plasticity (depressing and facilitating types) were used. Synaptic conductance values were constrained by performing in silico paired recordings to match experimentally measured postsynaptic potential (PSP) amplitudes. Parameters of the Tsodyks-Markram model for short-term synaptic plasticity were fitted using available thalamic data. Gap junction (GJ) connectivity between reticular neurons was predicted by dendrodendritic appositions. The density of GJs was adjusted to match experimental data on neuron divergence. Functionally, GJs were modeled as conductances coupling the membrane potential of adjacent compartments, and their conductance values were validated by comparing coupling coefficients with experiments. The model was validated at multiple levels by comparing its properties and behavior with experimental measurements that were not used during the model building steps.

#### Updating electrical models in the microcircuit

The original electrical models of^7^ were optimized with experimental data and BluePyOpt optimization software. Among their five electrical types, we replaced only the two models of TRN cells (cNAd and cAD, low threshold bursting models) with our own hoc files for Ecel1, Spp1, and Runaway, or any other variants. When pairing electrical models with a morphology reconstruction from the population of morphologies of,^7^ we checked for consistency of electrical features and confirmed no significant deviations from experimental data (see Figure 5). For mixed circuits, composed of more than one TRN electrical type, the morphology to couple with an electrical model was randomly assigned, following the desired cell composition. We then recalibrated the holding and threshold currents per neuron model, needed for certain protocols in the network simulations. We finally checked that the postsynaptic potentials were unaltered by the change of electrical models by running a validation similar to one for constraining synapse conductance values described in^7^ (not shown).

#### Spindle-like activity in the microcircuit

Spindle-like oscillations in the model were defined as in,^7^ characterized by: frequency within the *in vivo* spindle range, a waxing and waning amplitude envelope, generation through thalamoreticular interactions involving rebound bursting, and emergence during cortical up states mimicking sleep. Spindle activity arose from the interplay of intrinsic neuronal mechanisms and synaptic interactions between TRN and TC neurons. The spindle oscillation cycle was driven by cortical input, which triggered TRN activity and subsequent hyperpolarization in TC neurons. This often resulted in post-inhibitory rebound bursts in TC neurons, which then excited TRN neurons, repeating the process. The waxing phase of spindle oscillations resulted from the progressive recruitment of TRN neurons. The waning phase was attributed to short-term synaptic depression, leading to decreased postsynaptic potential amplitudes, and the buildup of mutual inhibition among recruited TRN neurons, acting as a self-limiting mechanism. GJ coupling between TRN neurons also significantly influenced spindle duration, synchronizing activity and transmitting low-threshold bursts across the network.

### Quantification and statistical analysis

For correlation analyses, we used the Pearson correlation coefficient (r) and the coefficient of determination $R^{2}$ to measure the relationship between variables.

## Footnotes

Supplemental information can be found online at https://doi.org/10.1016/j.isci.2025.113393.
