# Supplementary material for: Biophysical modeling of thalamic reticular nucleus subpopulations and their differential contribution to spindle dynamics
Source: iScience. 2025 Aug 20;28(9):113393. doi: 10.1016/j.isci.2025.113393 (PMC12454877; doi:10.1016/j.isci.2025.113393)
Supplement: Document S1. Figures S1–S10 and Tables S1 and S2 [file mmc1.pdf]

iScience, Volume *28*

Supplemental information

Biophysical modeling of thalamic reticular nucleus subpopulations and their differential contribution to spindle dynamics

Polina Litvak, Nolan D. Hartley, Ryan Kast, Guoping Feng, Zhanyan Fu, Alexis Arnaudon, and Sean L. Hill

1 Supplementary tables

feature name	mean	std
spike_count (holding at $-80mV$)	0.0	0.001
spike_count_start	0.0	0.001
voltage_std_start	0.01	0.05
burst_runaway*	0.2	100.0
tonic_after_burst*	5.0	10.0
burst_number *	5.0	100.0
spikes_per_burst	3.0	1.0
burst_mean_freq	150.0	150.0
peak_voltage	20.0	5.0
inv_first_ISI	150.0	20.0
AP2_AP1_peak_diff	-4.0	1.0
AHP_depth_abs	-70.0	5.0
time_to_last_spike*	10000.0	100000.0
time_to_first_spike	100.0	100.0
postburst_min_values	-87.0	10.0
maximum_voltage_from_voltagebase* (Ca^{2+} at $50\mu m$)	1.0	10.0
maximum_voltage_from_voltagebase* (Ca^{2+} at $150\mu m$)	1.0	10.0
maximum_voltage_from_voltagebase* (Ca^{2+} at $250\mu m$)	1.0	10.0

Table S1: Electrical feature names (as in eFEL, see `https://github.com/BlueBrain/eFEL`) and statistics (mean and standard deviation). Features with a * have a large std and can be considered as not constraining the MCMC sampling (as the cost was defined as the largest z-score).

Mechanism name	Added to	Bounds	Ecel1	Spp1	Runaway	Ecel1/Spp1/Runaway
Temperature (T)	-	-	25	25	25	34
Passive conductance (g_pas)	soma/basal	$[0.5e-5, 1.0e-4]$	7.58e-05	4.194e-05	3.523e-05	3.0e-05
Voltage shift of I_T (vshift_it2)	soma/basal	$[-10, 10]$	-1.69	-4.13	-5.88	-3.0
Fast transient Na (hh2_Na)	soma	$[0, 4.0]$	2.88	1.17	1.5	2.0
Delayed K (hh2_K)	soma	$[0, 3.0]$	0.76	1.28	1.60	1.0
Low-threshold Ca^{2+} (I_T)	soma	$[0.0, 0.001]$	0.00092	0.00088	0.00058	0.0005
Slope low threshold Ca^{2+} density (it2_slope)	basal	$[10.0, 100.0]$	517.16	357.01	418.0	300.0
Ca^{2+} -dependent K (I_{AHP})	soma	$[0.0, 0.08]$	0.0701	0.0324	0.0285	0.03
Slow Ca^{2+} dependent nonspecific cation (I_{CAN})	basal	$[0.0, 0.0002]$	6.031e-05	9.689e-05	10.02e-05	4.0e-05
Ca^{2+} -dependent K (I_{AHP})	basal	$[0.0, 0.015]$	0.000736	0.0138	0.0149	0.015
Low-threshold Ca^{2+} (I_T)	basal	$[0.0, 0.0015]$	0.000667	0.00077	0.00072	0.0005/0.0007/0.001
Transient voltage-gated K (I_A)	basal	$[0.0, 0.005]$	0.00144	0.0043	0.00195	0.00

Table S2: Parameter values of models and bounds used in the main text. The models at $25C$ are from MCMC, and the ones at $34C$ are for the circuit simulations (only the I_T in basal dendrites differs between them).

2 Supplementary figures

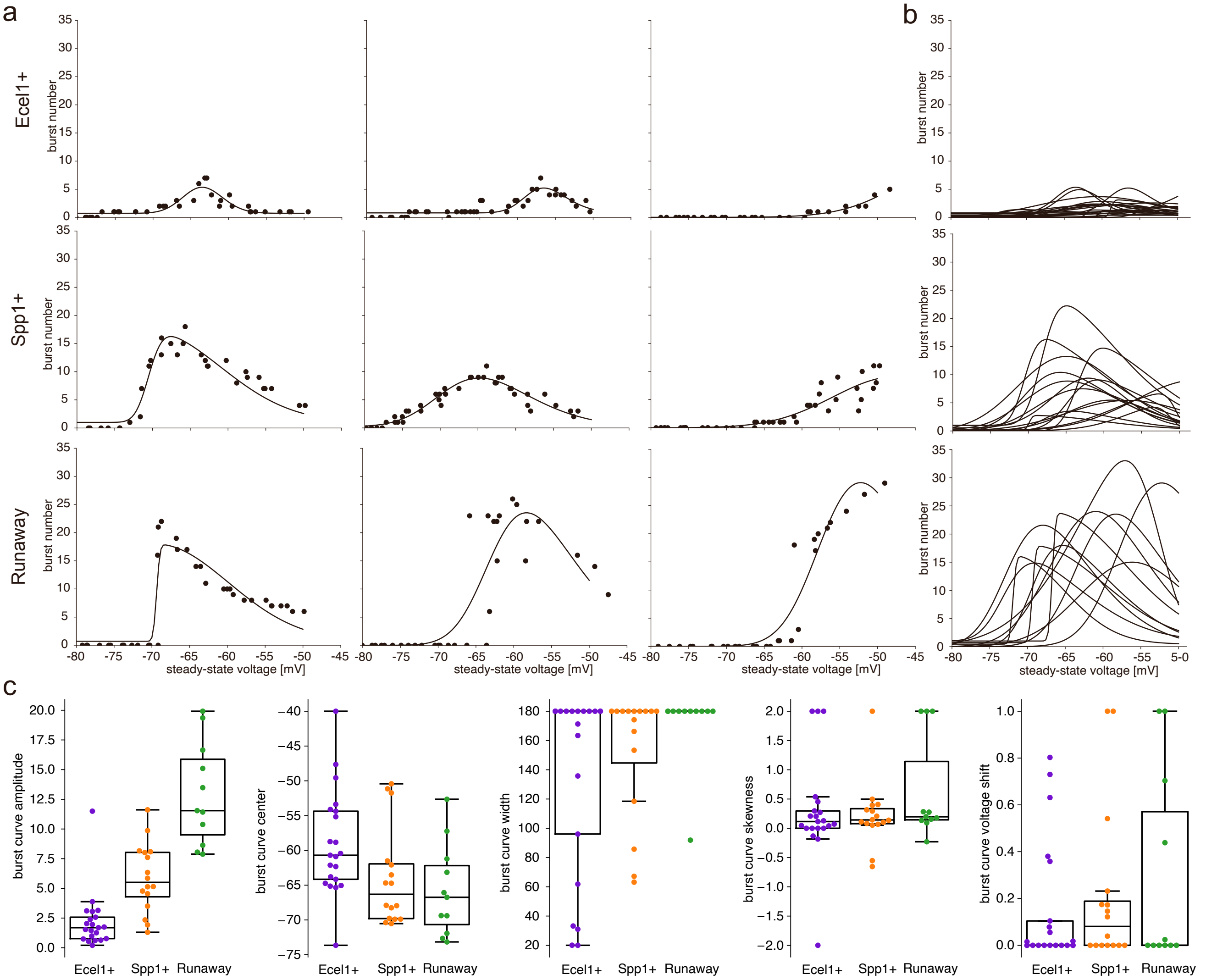

Fig. S1: **Variability in experimental burst curves a.** Three examples of experimental burst curves per cell type, illustrating cases with large skewness, or centered at large or low steady-state voltages. **b.** Superimposed fits of all burst curves, showing large variability per cell type. **c.** Fitted burst curve parameter distributions, by cell type.

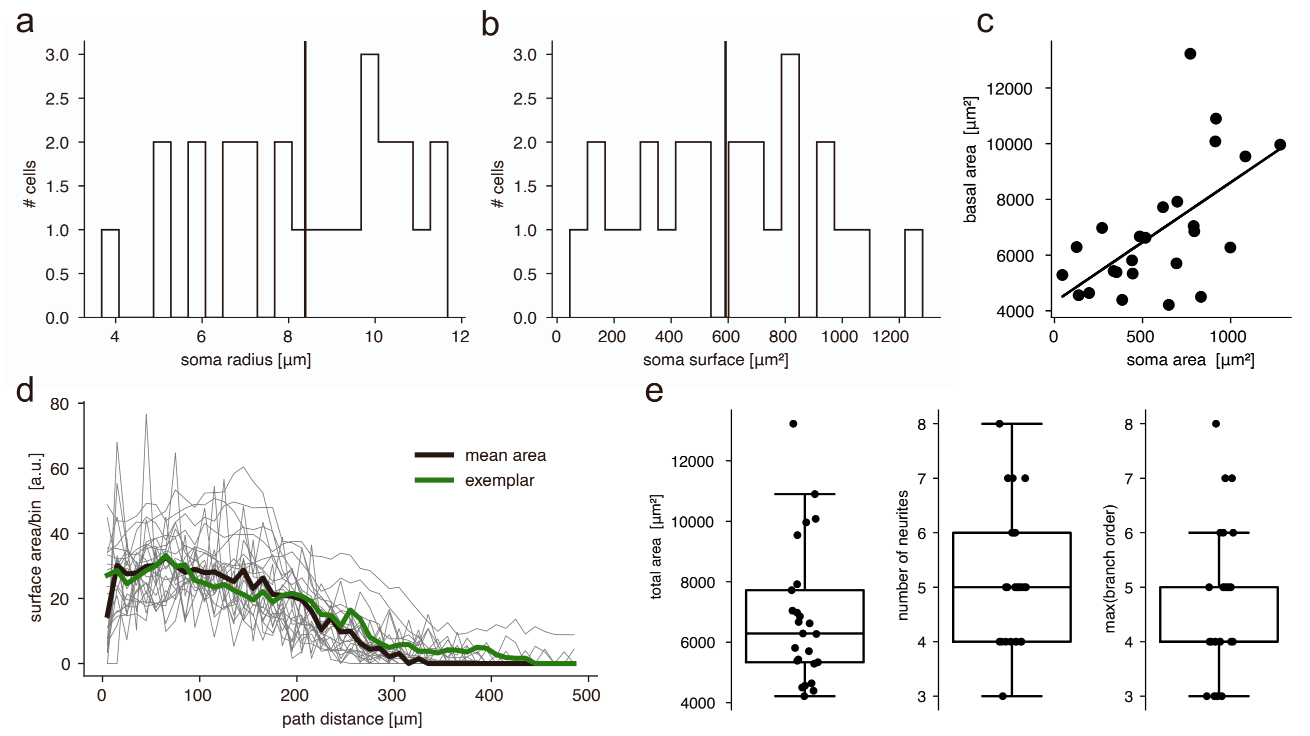

Fig. S2: **Morphological population a.** Distribution of soma radii from the population of morphologies of [1] **b.** Distribution of soma surface area from the same population. **c.** Correlation between soma surface area and basal surface area (Pearson r = 0.6). **d.** Surface area profiles of each morphology, their mean area and a chosen exemplar, closest to the mean. **e** Distribution of three morphometrics computed on this population: total surface area, number of neurites and maximum branch order.

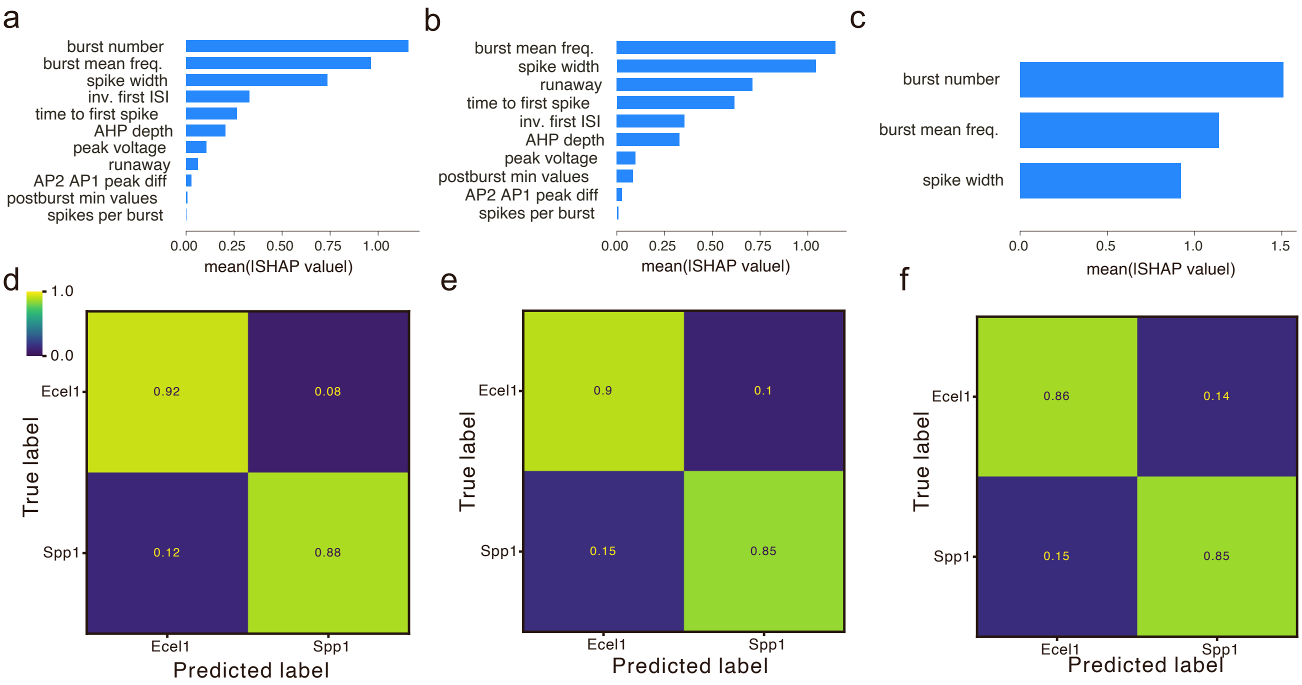

Fig. S3: **Cell type classification using electrical features a.** SHAP values for a 10-fold classification using all electrical features extracted from the rebound burst protocol voltage traces (accuracy of $88.2 \pm 13.7\%$) **b.** SHAP values for a 10-fold classification using electrical features as in a., but with maximum burst number per holding membrane voltage feature excluded ($86.0 \pm 15.7\%$) **c.** SHAP values for a 10-fold classification using the top three most influential electrical features ($87.6 \pm 13.8\%$) **d-f.** Confusion matrices for **a-c.** respectively. The confusion matrix was normalized by true values, and averaged over all training data (10 repeats of 10-folds).

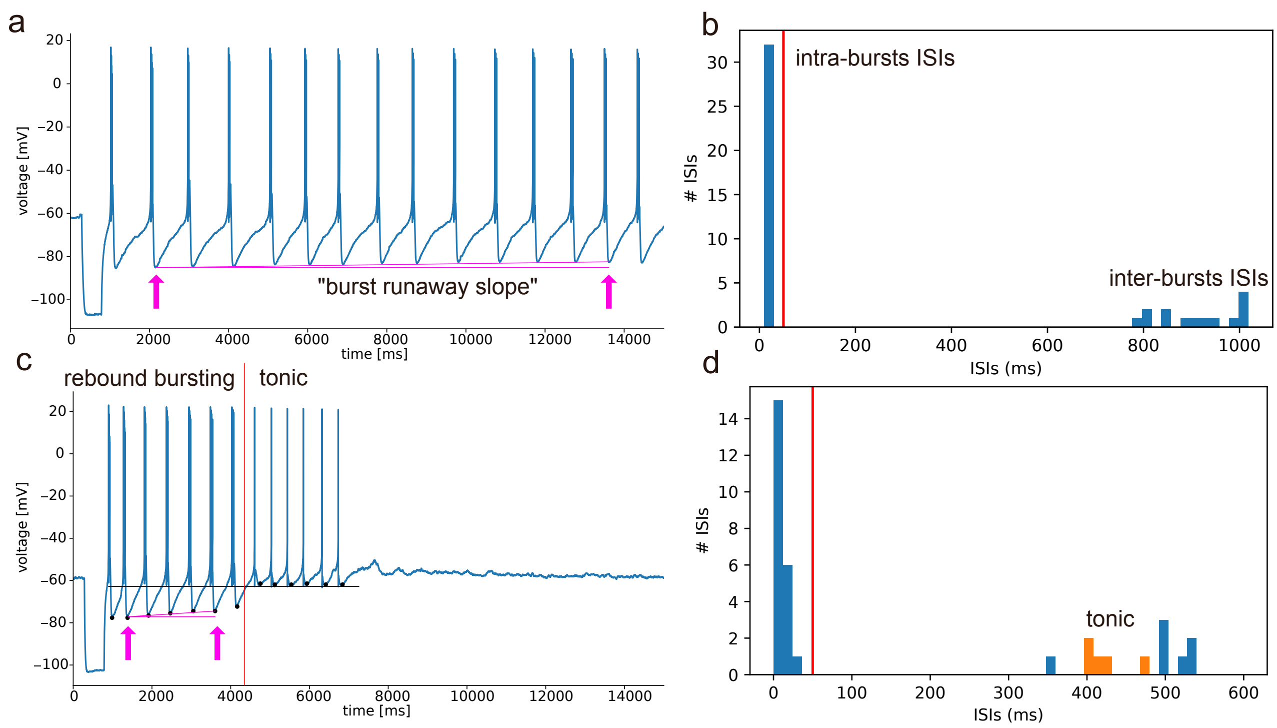

Fig. S4: **Electrical features for rebound burst characterisation a.** Rebound burst trace with first and last burst used to compute the runaway slope marked with arrows. The slope is computed from the slow AHP depths between these two bursts, assuming linearity. **b.** Inter-spike interval distributions of the trace in panel a, showing the small ISI within the bursts, and larger ones between bursts, defining the number of bursts (minus one). **c.** Trace of a cell rebound bursting, then transitioning to post-burst tonic spiking. Our runaway slope computation is also highlighted in panel a. Black dots mark slow AHP depths between bursts, and the black line shows fast AHP depths between APs in the bursts. What is detected as bursts with slow AHP depths above the mean value of the fast AHP depth are instead considered as post-burst tonic APs. **d.** From the split of bursts and tonic firing in panel c, we can differentiate the ISIs between rebound bursting and tonic firing and estimate the number of bursts and post-burst tonic APs.

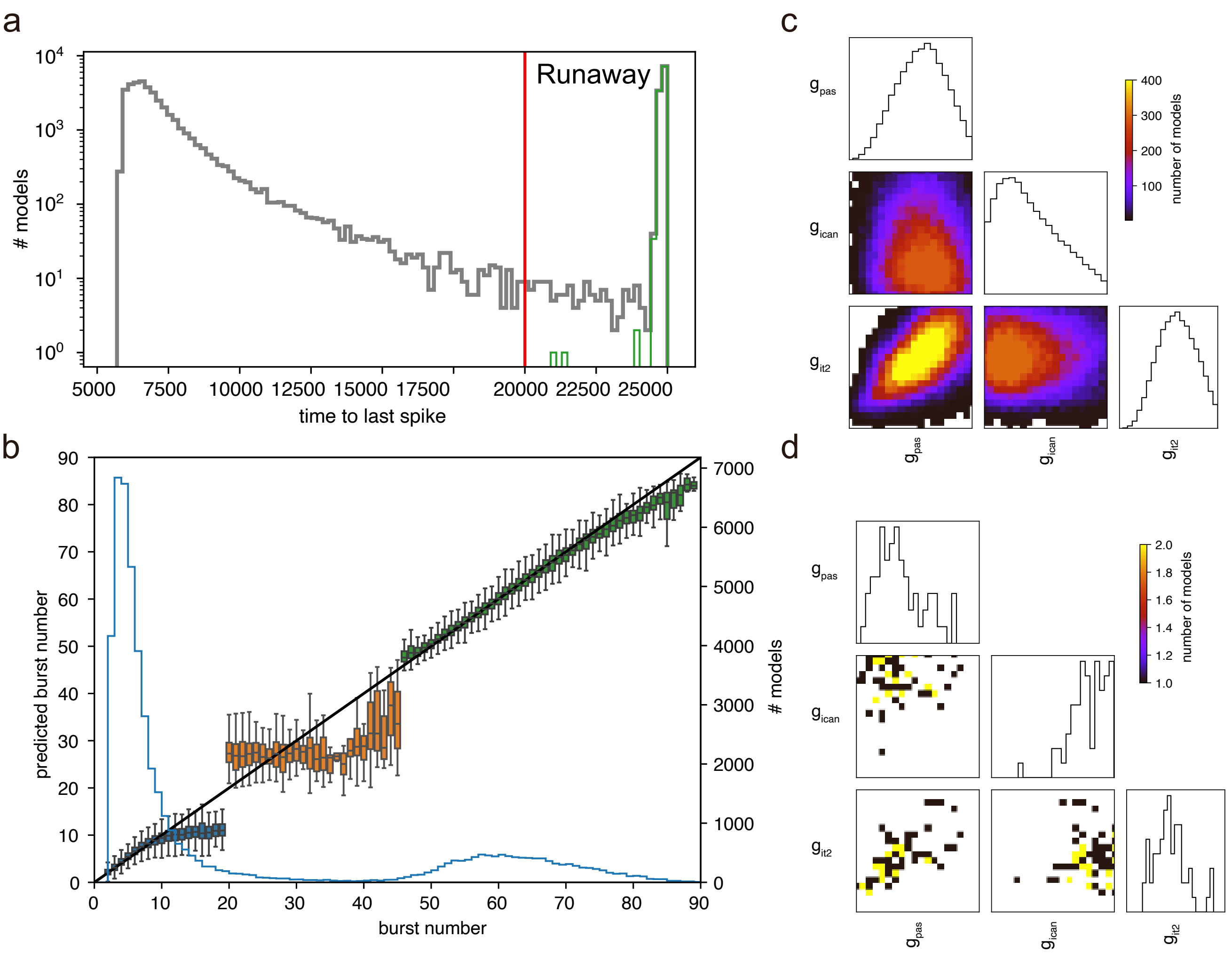

Fig. S5: **Additional panels for Fig 2 a.** Distribution of time to last spike with all models in gray, and Runaway in green. The red line is the threshold used to isolate Runaway models. **b.** Predicted burst number of an XGBoost regression in three parts, for low, middle and high burst numbers. A single model fails to fit the entire range, but a model fitted only to low or high range of burst numbers works. The blue line represents the density of models, and the errorbars the predictions. **c.** Sub-corner plot of three main parameters for post-burst tonic firing, for all models **d.** Sub-corner plot of the same parameters as in panel c, but only for models with post-burst tonic discharge present.

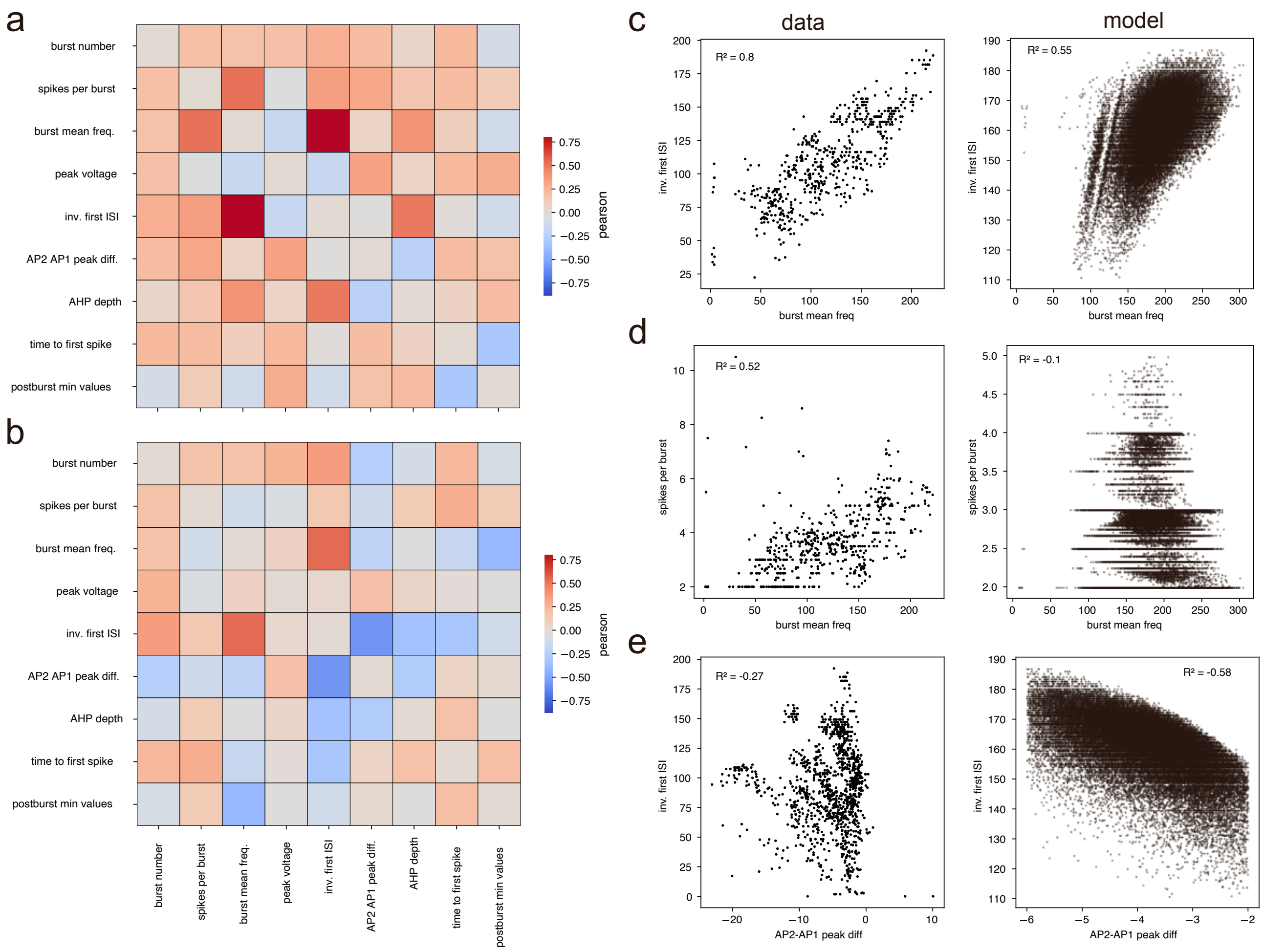

Fig. S6: **Two dimensional correlations between features in experimental data and models. a.** Pearson correlations of experimental data **b.** Pearson correlations of MCMC models **c-e.** Scatter plot of some strong correlations in data (left panels) and models (right panels)

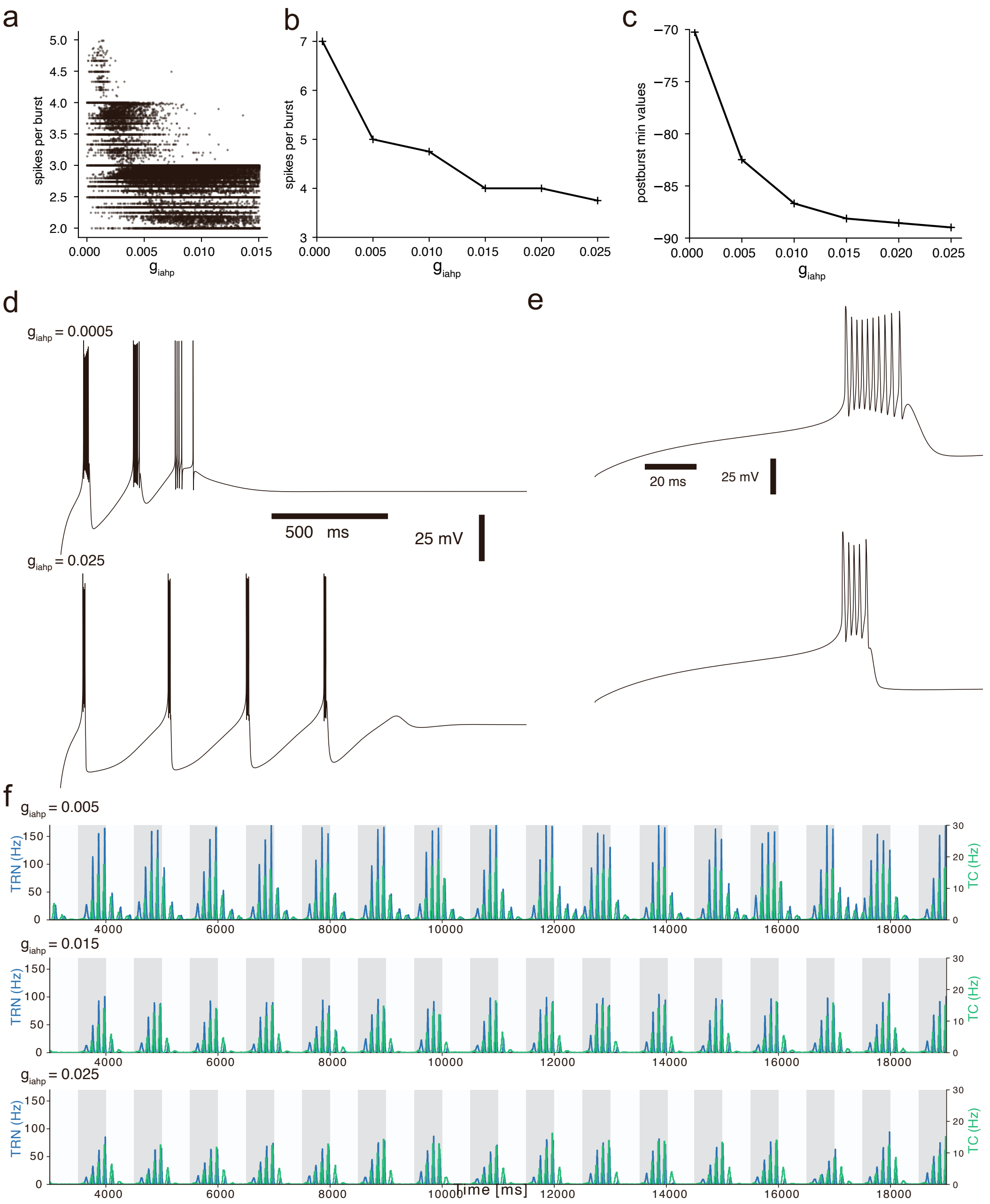

Fig. S7: **Effect of I_{AHP} conductance manipulations a.** Correlation between I_{AHP} conductance and number of APs per bursts in all MCMC models with cost < 2 std. **b.** Spikes per burst as a function of I_{AHP} in the Spp model. **c.** Average Slow AHP depth between bursts as a function of I_{AHP} . **d.** Traces of models with lowest and highest I_{AHP} values from the previous panel. **e.** Zoom on the first burst of traces in panel d. **f.** Population response PETH for three circuits with varied I_{AHP} conductance values. Middle row is the Spp1 model studied in Fig. 7i. Simulated cortical up states at 90 % CT input shown in gray.

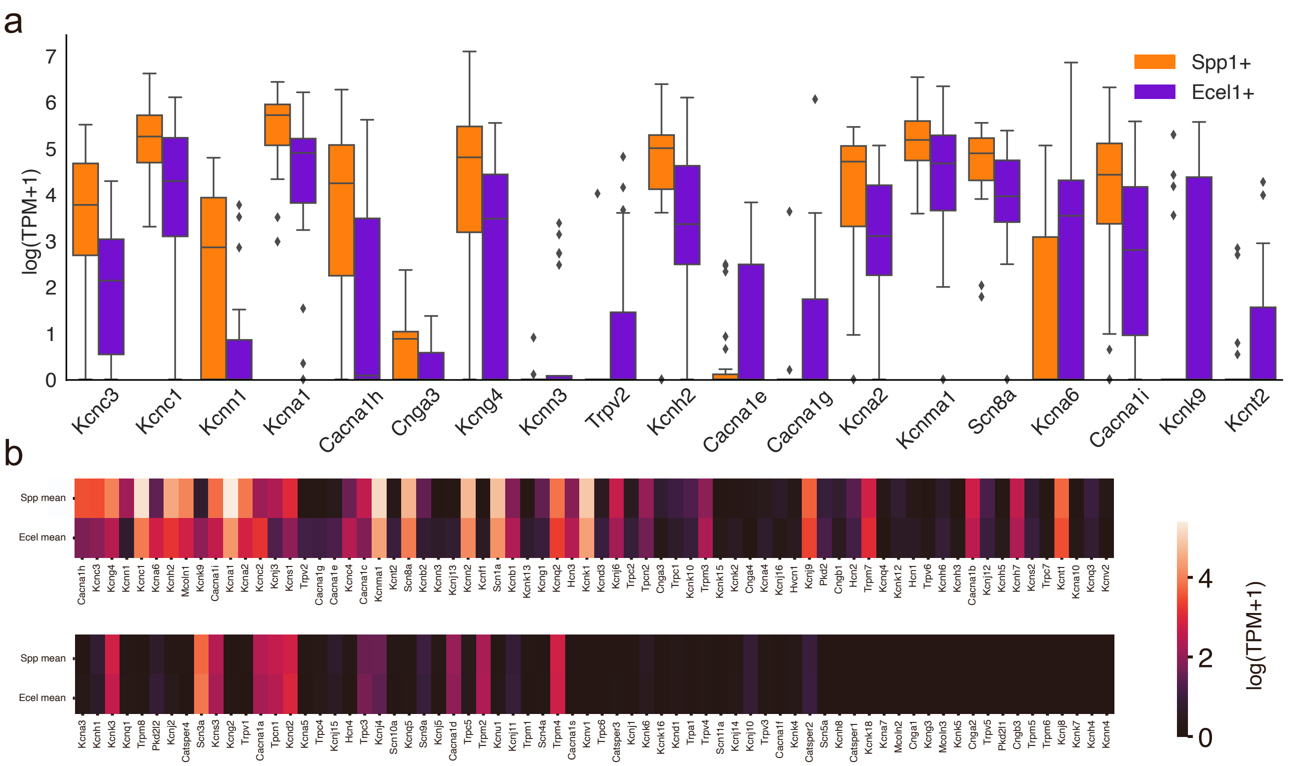

Fig. S8: **Single-cell transcriptomics data from [2] relevant for electrophysiological properties of Ecel1+ and Spp1+ neurons a.** Mean gene expression levels of the most differentially expressed genes.(TPM is Transcripts Per Million) **b.** Mean gene expression levels for each cell subtype, ordered by absolute difference.

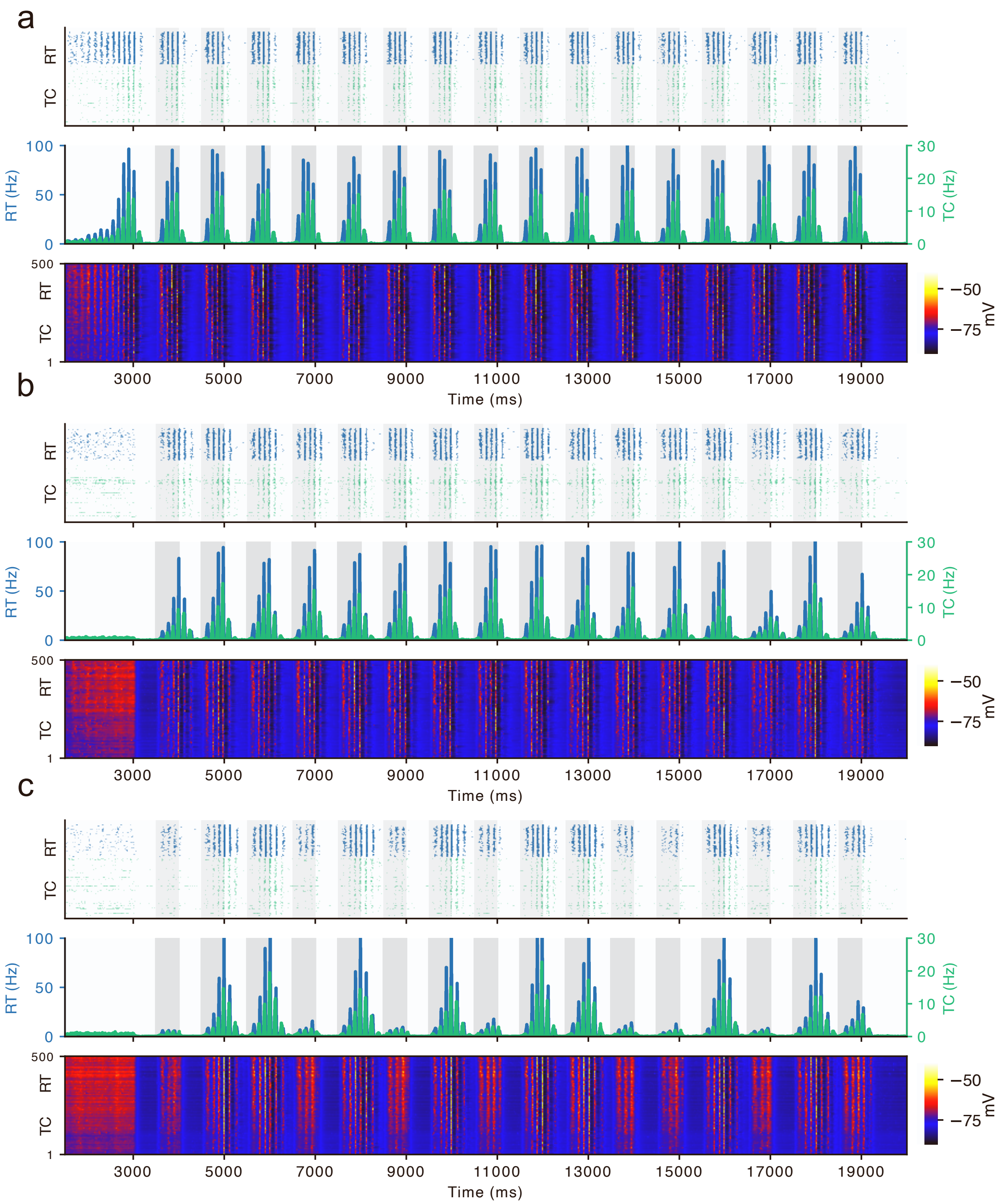

Fig. S9: **Population response spike raster, PETH and voltage raster of a subset of mixed composition circuits from Fig. 7b. a.** Population response in a mixed circuit composed of 50% Spp1 and 50% Runaway models. **b.** Population response in a mixed circuit composed of 50% Ecel1, 25% Spp1 and 25% Runaway models. **c.** Population response in a uniform circuit composed of Ecel1 models exclusively. Simulated cortical up states at 90 % CT input shown in gray

.

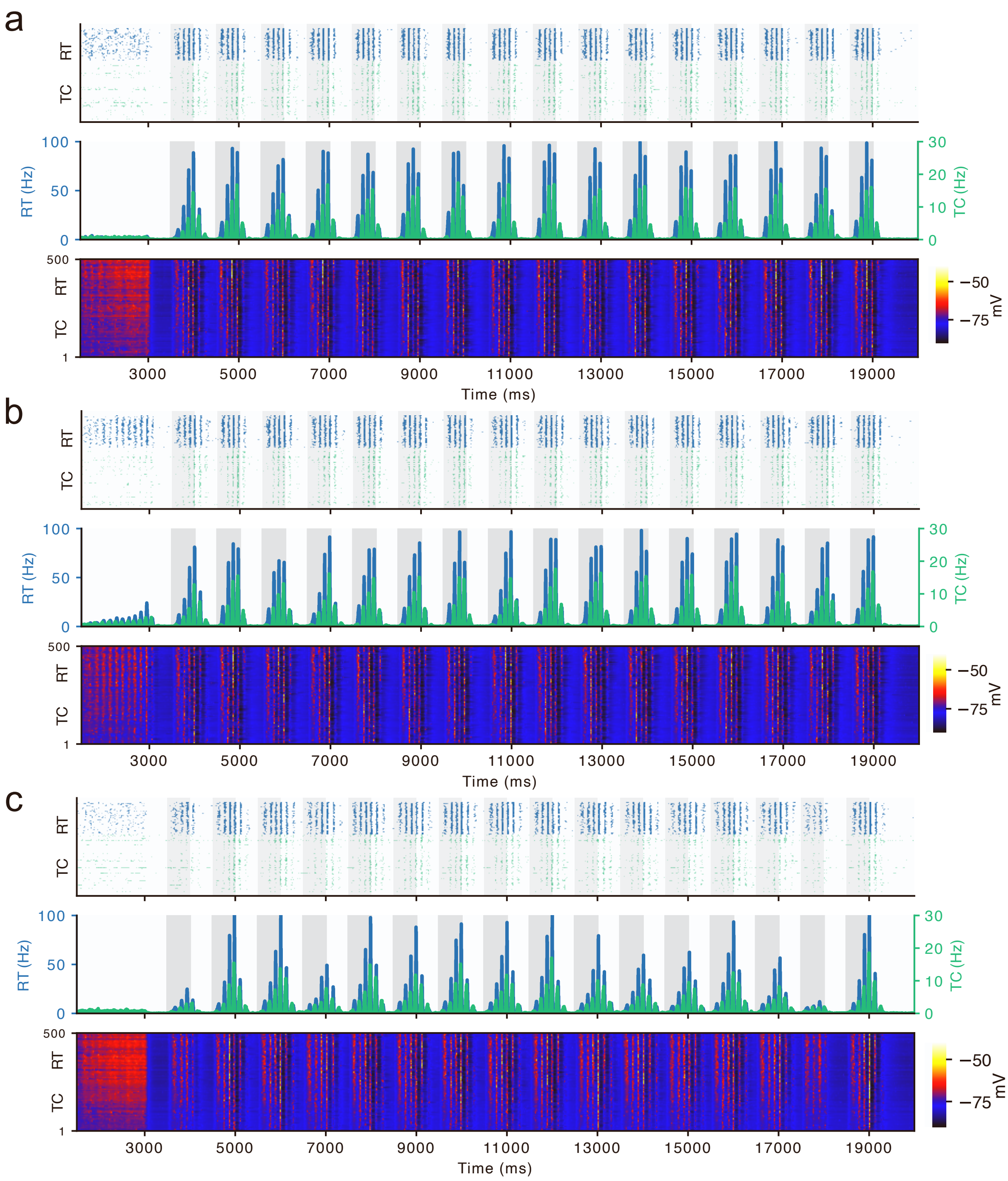

Fig. S10: **Population response spike raster, PETH and voltage raster of the circuits in Fig. 7f, reproducing** [2]**Fig. 5 a.** Population response in a mixed circuit composed of 10% Ecel1, 45% Spp1, and 45% Runaway models. **b.** Population response in a mixed circuit as in panel a, but with reduced I_T in Ecel1 models (marked with a magenta cross in the main figure). **c.** Population response in a mixed circuit as in panel a, but with reduced I_T in Spp1 and Runaway models (marked with a blue cross in the main figure). Simulated cortical up states at 85 % CT input shown in gray.

References

[1] Elisabetta Iavarone, Jane Simko, Ying Shi, Marine Bertschy, María García-Amado, Polina Litvak, Anna-Kristin Kaufmann, Christian O'Reilly, Oren Amsalem, Marwan Abdellah, et al. Thalamic control of sensory processing and spindles in a biophysical somatosensory thalamoreticular circuit model of wakefulness and sleep. *Cell Reports*, 42(3), 2023.

[2] Yinqing Li, Violeta G Lopez-Huerta, Xian Adiconis, Kirsten Levandowski, Soonwook Choi, Sean K Simmons, Mario A Arias-Garcia, Baolin Guo, Annie Y Yao, Timothy R Blosser, et al. Distinct subnetworks of the thalamic reticular nucleus. *Nature*, 583(7818):819–824, 2020.
